# New Aspects of Corpus Luteum Regulation in Physiological and Pathological Conditions: Involvement of Adipokines and Neuropeptides

**DOI:** 10.3390/cells11060957

**Published:** 2022-03-10

**Authors:** Ewa Mlyczyńska, Marta Kieżun, Patrycja Kurowska, Monika Dawid, Karolina Pich, Natalia Respekta, Mathilde Daudon, Edyta Rytelewska, Kamil Dobrzyń, Barbara Kamińska, Tadeusz Kamiński, Nina Smolińska, Joelle Dupont, Agnieszka Rak

**Affiliations:** 1 Laboratory of Physiology and Toxicology of Reproduction, Institute of Zoology and Biomedical Research, Jagiellonian University in Krakow, 30-387 Krakow, Poland; ewa.mlyczynska@doctoral.uj.edu.pl (E.M.); patrycja.kurowska@uj.edu.pl (P.K.); monika.dawid@doctoral.uj.edu.pl (M.D.); karolina.pich@doctoral.uj.edu.pl (K.P.); natalia.respekta@doctoral.uj.edu.pl (N.R.); 2 Department of Animal Anatomy and Physiology, Faculty of Biology and Biotechnology, University of Warmia and Mazury in Olsztyn, 10-719 Olsztyn, Poland; marta.kiezun@uwm.edu.pl (M.K.); edyta.rytelewska@uwm.edu.pl (E.R.); barbara.kaminska@uwm.edu.pl (B.K.); tkam@uwm.edu.pl (T.K.); nina.smolinska@uwm.edu.pl (N.S.); 3 Unité Physiologie de la Reproduction et des Comportements, French National Institute for Agriculture, Food, and Environment, 37380 Nouzilly, France; mathilde.daudon@inrae.fr (M.D.); joelle.dupont@inrae.fr (J.D.); 4 Department of Zoology, Faculty of Biology and Biotechnology, University of Warmia and Mazury in Olsztyn, 10-719 Olsztyn, Poland; kamil.dobrzyn@uwm.edu.pl

**Keywords:** corpus luteum, progesterone, luteinization, luteolysis, adipokines, neuropeptides

## Abstract

The corpus luteum is a small gland of great importance because its proper functioning determines not only the appropriate course of the estrous/menstrual cycle and embryo implantation, but also the subsequent maintenance of pregnancy. Among the well-known regulators of luteal tissue functions, increasing attention is focused on the role of neuropeptides and adipose tissue hormones—adipokines. Growing evidence points to the expression of these factors in the corpus luteum of women and different animal species, and their involvement in corpus luteum formation, endocrine function, angiogenesis, cells proliferation, apoptosis, and finally, regression. In the present review, we summarize the current knowledge about the expression and role of adipokines, such as adiponectin, leptin, apelin, vaspin, visfatin, chemerin, and neuropeptides like ghrelin, orexins, kisspeptin, and phoenixin in the physiological regulation of the corpus luteum function, as well as their potential involvement in pathologies affecting the luteal cells that disrupt the estrous cycle.

## 1. Introduction

The corpus luteum (CL) is a transient endocrine gland with a short lifespan including its development, functional establishment, and regression. The main function of CL is progesterone (P_4_) production, which prepares the uterus for implantation and pregnancy maintenance [1]. Besides, the luteal P_4_ content appears to provide a good index of normal CL function [2]. In the case of failed fertilization, CL regresses in the process of luteolysis, and subsequently, a new cycle may begin; hence, the CL also plays a central role in the regulation of the estrous/menstrual cycle [1]. Thus, the complex processes, including the formation, maintenance, and regression of CL, as well as efficient steroidogenesis, are among the most significant and strictly regulated by luteotropic and luteolytic factors/events in mammalian reproduction. Therefore, any abnormalities in the CL physiology may lead to numerous pathologies, and consequently, infertility. For instance, inappropriate vascularization leads to aberrant CL development and the reduced concentration of P_4_ named the luteal phase deficiency [3]. These disturbances have a negative impact on endometrium growth and its secretory activities, causing miscarriages or preterm delivery [4,5]. On the other hand, the overstimulated proliferation and luteinization of anovulated follicle, without ovulatory luteinizing hormone (LH) peak, leads to CL cyst formation [6], whereas stromal luteoma [7] and pregnancy luteoma are CL tumors connected to the extensive proliferation of large luteal cells (LLCs) [8]. Thus, knowledge about new factors participating in the CL formation and modulation of such processes as steroidogenesis, angiogenesis, cell proliferation, and apoptosis, as well as the mechanism of its actions and interactions, is necessary to prevent infertility events connected with CL dysfunction.

Reproductive success depends on physiological mechanisms that control hormonal homeostasis influencing female reproduction on four levels: central effects on the hypothalamus and pituitary, peripheral and local effects on the ovary and reproductive tract, direct effects on the oocyte and embryo, and effects during pregnancy. Thus, in recent years, a lot of attention has been paid to new endocrine factors like neuropeptides, as well as adipokines produced by the white adipose tissue (WAT) which influence the hypothalamic–pituitary–ovarian (HPO) axis [9]. The expression of several adipokines including adiponectin, chemerin, resistin, visfatin, omentin, as well as their receptors has been described both in human and animal reproductive tissues. The adipokines were found to regulate female fertility by direct influence on the numerous processes, including oocytes maturation, follicular steroidogenesis, angiogenesis, cell proliferation, and apoptosis, as well as fertilization, implantation, and placental function [10]. Moreover, neuropeptides, like phoenixin, which affects pituitary hormones secretion, regulates the estrous cycle, ovarian follicles maturation, and ovulation [11], or kisspeptin (Kiss1), which controls Gonadotropin-releasing hormone (GnRH) secretion, mediate endocrine and metabolic inputs into the regulation of human reproduction [12].

In the present review, we described the following adipokines: leptin, adiponectin, apelin, visfatin, vaspin, chemerin, as well as neuropeptides: orexins, ghrelin, Kiss1, and phoenixin impact on luteal cells physiology, focusing on its formation, angiogenesis, steroidogenesis, prostaglandin synthesis, proliferation/apoptosis, and regression processes. In this paper, we also analyzed their connection with CL pathologies such as cysts formation, luteal dysfunction, or neoplasia, and postulated their future implications in pathologies recognition or treatment.

## 2. Corpus Luteum Structure and Physiology

The corpus luteum develops immediately after ovulation by forming from the ovarian follicle cells in a process called luteinization. It is a heterogeneous structure composed mainly of two types of steroidogenic cells [13]. LLCs come from granulosa cells (Gc), while small luteal cells (SLCs) have theca interna cells (Tc) origin. Besides this, the CL includes other types of cells, such as pericytes [14] and endothelial cells [15], as well as fibroblasts [16], and cells of the immune system (macrophages, lymphocytes, neutrophils) [17]. These cells are also sources of hormones, growth factors, and cytokines, and their mutual interactions are important in both luteinization and luteolysis. In the first days of the luteal phase, after ovulation, the follicle walls collapse, and the basement membrane between the Gc and Tc disappears. In this way, blood vessels can penetrate the developing CL. The above-described luteinization is typical and occurs, for example, in pig. In humans, SLCs do not mix with LLCs and form an outer layer surrounding LLCs. In some species, such as horses and marmosets, CL is composed entirely of cells derived from Gc [13]. During luteinization occurs hypertrophy, which causes an increase in the size of the gland. Generally, in luteal cells (LCs), the suppression of cell proliferation and their arrest in the G1 phase are observed. Cell cycle inhibitors, such as cyclin-dependent kinase inhibitor 1B (p27Kip1), are expressed in LCs. Nevertheless, in some species like pigs and sheep, LCs derived from the Tc retain their ability to proliferate [18]. The developing CL so-called corpus hemorrhagicum requires the creation of blood vessels to ensure proper blood flow to the gland and transport of hormones. The endothelial cells and pericytes present in the CL are responsible for the formation of blood vessels. Endothelial cells produce angiogenic factors such as vascular endothelial growth factor (VEGF), fibroblast growth factor (FGF), angiopoietin 1 (ANG-1), and many others, which in turn stimulate their proliferation and migration, thus creating blood vessels [19].

**Figure 1:**
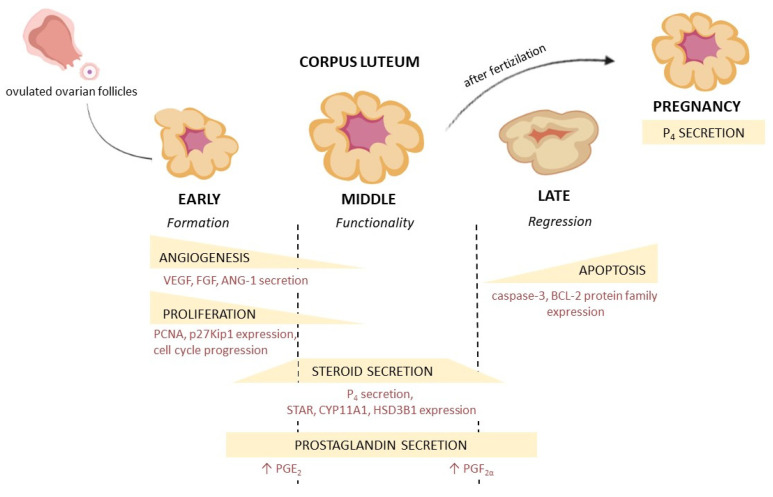
Corpus luteum development, morphological and hormonal changes during the luteal phase. VEGF—vascular endothelial growth factor, FGF—fibroblast growth factor, ANG-1—angiopoietin 1, PCNA—proliferating cell nuclear antigen, p27Kip1—cyclin-dependent kinase inhibitor 1B, P_4_—progesterone, STAR—steroidogenic acute regulatory protein, CYP11A1—cytochrome P450 family 11 subfamily A member 1, HSD3B1—hydroxy-delta-5-steroid dehydrogenase, BCL-2—B-cell lymphoma-2 protein, PGE_2_—prostaglandin E2, PGF_2α_—prostaglandin F2α.

The main function of the CL is the already mentioned production of P_4_ which enables embryo implantation and the maintenance of pregnancy (Figure 1). Basically, both SLCs and LLCs, are capable of producing this steroid, however, LLCs are more secretory-active. They are characterized by regular, spherical cell nuclei and an extensive cytoplasm. In addition, LLCs have a greater rough and smooth endoplasmic reticulum, as well as the Golgi apparatus, numerous mitochondria, and lipid droplets, and regardless of the LH level, they produce large amounts of P_4_. Thanks to the occurrence of rough endoplasmatic reticulum and unlike SLCs, LLCs are able to produce peptide hormones and cytokines. In turn, SLCs have irregular nuclei and a less developed endoplasmic reticulum. Unlike large cells, they remain dependent on LH stimulation, and when this hormone reaches high levels in the blood, they are able to produce P_4_. After LH binds to its receptor on the surface of these cells, adenylate cyclase is activated, the cyclic adenosine monophosphate (cAMP) levels increase, and the protein kinase A (PKA) pathway is activated, which ultimately results in an increase in P_4_ production [20]. The most important step in luteal P_4_ production is the transport of cholesterol to the inner mitochondrial membrane. Steroidogenic acute regulatory protein (STAR) is a factor that is responsible for this process [21]. Subsequently, the cytochrome P450 family 11 subfamily A member 1 (CYP11A1), located in the mitochondrial membrane, catalyzes the conversion of cholesterol to pregnenolone (P_5_), which diffuses out the mitochondria. The final stage takes place in the smooth endoplasmic reticulum where P_5_ is converted to P_4_ thanks to the activity of the hydroxy-delta-5-steroid dehydrogenase (HSD3B1) [22].

The corpus luteum has a limited life span and must regress if the oocyte is not fertilized. The development of the CL, as well as its regression, occurs in a similar way in many species, but different factors are involved in the regulation of these two processes. The most important luteotropic factors in many species are LH (humans, ruminants, and pigs) [23], prolactin (PRL) (in rodents and rabbits) [5], and prostaglandin E_2_ (PGE_2_) (in humans, cattle, and ruminants) [24,25]. Luteolysis is mainly initiated by another prostaglandin—prostaglandin F2α (PGF_2α_), which is derived mostly from the uterus. In cattle and pigs, uterine PGF_2α_ additionally initiates local PGF_2α_ production in CL. Luteolytic changes appear around day 17 of the estrous cycle in cow, and on day 13 in pigs. On the surface of LCs, the expression of the prostaglandin F receptor (PTGFR) increases, and they become sensitive to PGF_2α_ [26]. Binding to the receptor results in the activation of phospholipase C, increasing the level of Ca^2+^ and, consequently, the activation of protein kinase C (PKC). Subsequently, P_4_ secretion is inhibited, autophagy together with apoptotic changes in CL appear, leading to its complete regression [20]. In primates, PGF_2α_ does not initiate luteolysis. The process is the result of insufficient LH stimulation [27]. However, if fertilization occurs, the cyclic CL becomes gestational CL, and produces P_4_ continuously until the end of pregnancy, as in pigs, cows, and dogs, or for part of it, as in humans and cats, where the gestational CL is functional only for the first part of pregnancy when its function is taken over by the placenta [28].

## 3. Characteristic of Adipokines and Neuropeptides, Their Receptors, and Mechanism of Action

### 3.1. Leptin

Leptin is a small 16 kDa protein of great physiological importance due to its pleiotropic function in various systems and tissues in humans and other species. The discovery of this hormone in 1994 initiated research into the endocrine role of the adipose tissue and resulted in the discovery of other adipokines. Leptin expression is mainly noted in the WAT and its level increases in obese people [29]. Therefore, circulating leptin levels change with the nutritional status and show circadian fluctuations [30]. The human leptin gene is located on chromosome 7 and encodes a 167 amino-acid (aa) product. In mammals, leptin’s aa sequence is highly conserved, and there are also orthologs in amphibians, reptiles, and fish [31]. Nevertheless, the duck and chicken leptin transcripts have only 26–30% of identity in common with human leptin [32]. The main characteristic function of leptin is to maintain energy metabolism from the central level in the brain, influencing the appetite and satiety center, to the peripheral action in muscles, pancreas, and liver, regulating glucose metabolism [33]. In mammals, leptin binds to its receptor (LEPR, also called OBR) to activate multiple signaling pathways as Janus kinase 2/signal transducer and activator of transcription 3 (JAK2-STAT3), mitogen-activated protein kinase (MAPK), phosphatidylinositol-4,5-bisphosphate 3-kinase/protein kinase B (PI3K/AKT), and protein kinase AMP-activated catalytic subunit alpha 2 (AMPK or PRKAA2) [34]. There are at least five leptin receptor isoforms because of alternative splicing (LEPRa, LEPRb, LEPRc, LEPRd, and LEPRe), which have the same *N*-terminal ligand-binding domain and a different *C*-terminal region. The LEPRa, LEPRb, LEPRc, and LEPRd have a single transmembrane region, while LEPRe (also called SLR for soluble leptin receptor) is truncated proximal to the membrane-spanning domain [35]. These isoforms are expressed in the major part of tissues [36]. The LEPRb is the longest isoform and is essential for energy homeostasis. Similarly, short OBR isoforms allow bodyweight regulation [35].

Concerning JAK2/STAT3 pathway, LEPR activation induces the recruitment and activation of JAK2 which phosphorylates tyrosine residues within the cytoplasmic domain of LEPRb and generates multiple signal cascades. This activates STAT proteins that play a role in the regulation of the transcription of genes important for food intake and lipid metabolism. In the hypothalamus, leptin inhibits AMPK, resulting in an increase in acetyl-coenzyme A carboxylase (ACC) activity and the reduction of food intake. In contrast, in mice skeletal muscles, leptin activates AMPK, causing a decrease in ACC and carnitine palmitoyltransferase 1 (CPT1) activity, and subsequently, inducing an increase in mitochondria β-oxidation [35] and fatty acid oxidation. Leptin can stimulate AMPK phosphorylation directly in skeletal muscle, but also indirectly via the hypothalamic-sympathetic nervous system axis [37]. Leptin can also inhibit the hepatic stearoyl-Coenzyme A desaturase-1 (SCD-1) activity to regulate lipoprotein metabolism and energy expenditure. In addition, leptin modulates, via the JAK/STAT pathway, the expression of genes important for thermogenesis, such as the thyrotropin-releasing hormone (TRH) in rats [35].

Leptin also plays a role in reproductive functions. Indeed, leptin gene knockout mice are infertile due to the incapacity of the hypothalamus to produce GnRH. This phenomenon is restored by exogenous leptin administration [34]. Leptin receptors are highly expressed in the hypothalamic-pituitary-adrenal (HPA) axis and gonads [32]. In mammals, it seems that leptin affects ovarian functions. Indeed, it has an antagonistic effect on in vitro insulin-like growth factor 1 (IGF1)-induced steroidogenesis in Gc and Tc, and enhances in vitro mammalian and avian oocytes’ cytoplasmic and nuclear maturation, as well as porcine embryo development [34,38].

### 3.2. Adiponectin

In 1996, Maeda et al. isolated, for the first time, adiponectin cDNA from the human WAT [39]. At the same time, Hu et al. isolated adiponectin cDNA from a murine fibroblast cell line [34,40]. Adiponectin is the most abundant hormone in the human plasma and the most abundant protein in human adipose tissue [34,41]. The human adiponectin gene contains three exons [42]. The full-length human adiponectin protein is composed of 244 aa. After proteolytic cleavage, a small fragment is generated: the globular domain of the protein (gAd) which is present in the plasma [43]. The primary structure of adiponectin is composed of an *N*-terminal collagen domain, a signal sequence of 18 aa, a variable region of 24 aa, a collagen-like fibrous domain of 65 aa, a C1q-like globular domain of 137 aa, and finally, of a *C*-terminal globular domain [44]. After translation, there is a modification of the structure by hydroxylation and glycosylation. Adiponectin can create three oligomeric isoforms: low molecular weight (LMW; 60 kDa) composed of three adiponectin monomers (28 kDa), middle molecular weight (MMW; 150 kDa), and high molecular weight (HMW; multimer of 12–32 adiponectin monomers) [42]. In chicken, the predominant isoform in plasma and adipose tissue is the HMW form. This phenomenon could be explained by the higher number of lysine residues in the chicken collagen domain compared to the human’s one, an element able to generate the multimerization and formation of a stable unique HMW isoform [45].

Adiponectin receptors are AdipoR1 and AdipoR2; there is a close homology between avian and mammalian AdipoR1 and AdipoR2, which may suggest that their genes are evolutionarily conserved [46]. They are composed of an *N*-terminal domain, seven transmembrane domains, and a *C*-terminal domain. However, they differ from G-protein coupled receptors, as their *N*-terminal region is cytoplasmic and the *C*-terminal region is extracellular [42]. AdipoR1 is abundant in skeletal muscles, while AdipoR2 is abundant in the liver [47]. Moreover, T-cadherin acts as a receptor of HMV and MMW, but not for LMW adiponectin isoforms in skeletal muscles [48]. The HMW isoform plays a significant role in the regulation of insulin signaling [43]. Adiponectin and its receptors were also found in turkey [49] and goose [50].

The main function of adiponectin is to improve insulin sensitivity and enhance glucose utilization and fatty acid oxidation [34]. Adiponectin could initiate the adaptor protein, phosphotyrosine interacting with the PH domain, and the leucine zipper 1 (APPL1)-AMPK signaling pathway. APPL1 binds to the intracellular domain of AdipoR and generates the translocation of transcription factors into the nucleus. This signaling induces cell migration, matrix metalloproteinases (MMP) activation, and collagen remodeling in the rat cardiac fibroblasts [51], and attenuates neuronal apoptosis in hypoxia-induced ischemia in neonatal rats [52]. Moreover, adiponectin-induced STAT3 phosphorylation and could be positively influenced by APPL1. This process generates an inhibition of the adiponectin effects on hepatic gluconeogenesis in male C57BL/6 mice. Finally, adiponectin, by the activation of p-AMPK and peroxisome proliferator-activated receptor α (PPARα) signaling pathways, promotes fatty acid oxidation. Adiponectin is also able to generate vasodilatation, endothelial cell proliferation, and migration via endothelial nitric oxide synthase (eNOS) phosphorylation induced by p-AMPK. The stimulation of glucose uptake by adiponectin is achieved through GLUT4 translocation mediated by the p-p38 MAPK [42]. In chickens, it was shown that adiponectin inhibited the lipid deposition and the differentiation of preadipocytes via p38 MAPK/ATF2 and TOR/p70 S6 kinase signaling pathways [53].

In the case of reproductive functions, it was shown that adiponectin is able to regulate in vivo and in vitro gonadotropin secretion and *GnRH* and *KISS1* genes expression. Indeed, adiponectin was shown to regulate ovarian steroidogenesis in most mammals [34]. In rats, adiponectin, AdipoR1, and AdipoR2 (commonly named as the adiponectin system) are strongly expressed in oocytes, cumulus cells, Tc, and less abundantly, in Gc. It has been hypothesized that adiponectin could be involved in Gc steroidogenesis [54]. In humans, it has been shown that Tc expressed adiponectin, AdipoR1, and AdipoR1, while Gc expressed both receptors. Adiponectin could have a potential implication in polycystic ovary syndrome (PCOS) [55]. In mice and humans, adiponectin supplementation during in vitro maturation has positive effects on early embryo development and meiotic progression [56]. In hens, adiponectin is more expressed in Tc than Gc from preovulatory follicles, AdipoR1 is more expressed in Gc than Tc, and AdipoR2 is expressed in the same way in both types of follicular cells. Thus, it seems that adiponectin could be involved in chicken preovulatory follicle development and oocyte maturation [34,57]. It has been shown that the adiponectin system is expressed at different stages of chicken embryo development [58].

### 3.3. Apelin

In 1998, during experiments focused on searching for a ligand for the G-coupled receptor (GPCR) APJ, Tatemoto et al. [59] purified apelin from bovine stomach extracts. The apelin cDNA encodes 77 aa preproproteins. In humans and cattle, the *N*-terminal end is rich in hydrophobic aa, suggesting that it is the secretory signal sequence. The aa sequence of the isolated bovine peptide corresponds to the deduced sequence of the preprotein from positions 42 to 58, suggesting that apelin is one of the processing products derived from the preprotein *C*-terminal end [59]. After post-translational modification, many active isoforms of apelin rise which are different in aa sequences length: apelin-36,-17, -13, and the last form of pyroglutamylated apelin-13 [60]. The distribution of each of the mature apelin forms in tissues is different [61].

APJ is a G protein-coupled receptor and it was identified in 1993 by O’Dowd et al. [62]. The gene of apelin receptor encodes a protein of 380 aa. The APJ contains seven hydrophobic transmembrane domains with consensus sites for phosphorylation by PKA, glycosylation, and palmitoylation [62]. In the Chinese hamster ovary (CHO) cells, apelin-13 and apelin-36 activate the ERK1/2 signaling pathway by binding to APJ coupled with Gi1 and Gi2. On the other hand, it has been shown that the activation of Akt kinase is a downstream effect of apelin signaling [63]. Furthermore, in human umbilical vein endothelial cells (HUVEC), apelin induces the double phosphorylation of the ribosomal protein S6 kinase B2 (p70S6K) generating cell proliferation through PTX-sensitive G-protein, ERK1/2, Akt, and mTOR cascades. Without apelin, APJ heterodimerizes with other GPCRs and is also able to activate signaling pathways [64].

The expression of the apelinergic system (apelin and its receptor) was found in human Gc and Tc, cumulus, and weakly in the oocyte [64], as well as in the Tc of mice [65]. In pig, the expression of the apelinergic system is observed in the ovarian follicles, and increases during follicle growth [66]. Apelin enhances estradiol (E_2_) and P_4_ secretion in human and porcine Gc [66,67]. In cattle, it has a negative effect on in vitro oocyte maturation by blocking the meiotic progression at the germinal vesicle stage [68]. Apelin enhances rat, porcine, and bovine granulosa cells proliferation [66].

### 3.4. Visfatin

Visfatin is a 52 kDa protein that is considered a cytokine, enzyme, and adipokine, resulting from its multidirectional action in the organism. It was first identified in 1994 when Samal et al. [69] cloned a cytokine called pre-B-cell colony enhancing factor (PBEF) from a human peripheral blood lymphocyte cDNA library. Another name that can be found in the literature for this protein is nicotinamide phosphoribosyltransferase (NAMPT). The human visfatin is predominantly expressed in bone marrow, muscles, and liver [69]. In 2002, Rongvaux et al. [70] found the murine homolog of PBEF. They characterized it as an enzyme catalyzing the reaction between nicotinamide and 5-phosphoribosyl-1-pyrophosphate to yield nicotinamide mononucleotide (NMN), being an intermediate in the nicotinamide adenine dinucleotide (NAD) biosynthesis [70]. At the same time, visfatin was identified as a cytokine hormone and an enzyme involved in immune and metabolic disorders [64]. In mammals, visfatin/NAMPT exists in 2 forms: extra- (eNAMPT) and intracellular (iNAMPT) [71]. The iNAMPT is a NAD biosynthetic enzyme and it plays an important role in the activation of sirtuin in mitochondria [72]. It is also involved in the metabolism, cellular regulation to nutrient availability, maturation, and cell survival [34]. In contrast, eNAMPT is released by cancer cells and could be used as a cancer-derived biomarker [72,73]. It also acts as an adipokine. Therefore, visfatin is involved in energy metabolism in mammals and birds [34]. In 2012, Li et al. [74] cloned the chicken visfatin gene from adult gonads and liver. The chicken visfatin protein has a high sequence identity in common with humans and rats [74]. In chickens, visfatin is expressed more strongly in skeletal muscles [75,76].

The visfatin receptor and its cellular mechanism of action remain unclear. However, some scientists have involved the insulin receptor signaling pathway in the visfatin action [77,78,79]. Moreover, the recently published data also indicate that visfatin can participate in inflammation processes by the activation of Toll-like receptor 4 (TLR-4) [80,81].

Visfatin expression was noted in different structures of ovaries in hens, turkeys, cattle, mice, and humans [49,82]. In cattle, visfatin is expressed in Tc and Gc, cumulus cells, and oocytes [83], while in mice, visfatin expression was noted additionally in stromal and endothelial cells [84]. In humans, visfatin is expressed in Gc, cumulus cells, oocytes, and, to a lesser extent, in Tc [85]. An in vitro study showed that visfatin inhibits P_4_ production in Gc via STAR and HSD3B downregulation [86]. Contrary to these reports in cows, it was shown that visfatin increases steroidogenesis and potentializes effects of IGF1 by increasing STAR and HSD3B expression and, consequently, E_2_ and P_4_ secretion [83]. In the male reproductive system, visfatin is expressed in human and rat testis [87,88] and human spermatozoa [87]. These findings implicate visfatin in rat spermatogenesis and steroidogenesis [88].

### 3.5. Vaspin

Vaspin, also named SerpinA12, belongs to the serine proteases inhibitors family [89], targeting kallikrein 7 and 14 [90,91]. This adipokine is encoded by the *SERPINA12* gene that is present on the long arm of chromosome 14 (14q32.1) in humans and consists of 1236 nucleotides [89]. The encoded protein is composed of three β-sheets, nine α-helices, and a flexible reactive center loop with a protease recognition sequence on the top. The signal peptide is a part of the N-domain [92]; additionally, at asparagine residues of this domain in humans, there are three predicted glycosylation sites [93]. Vaspin molecular weight is 45 kDa and the protein is composed of 392, 394, and 395 aa in rats, mice, and humans, respectively. There is 61.5% identity in common between human and rat vaspin aa sequences [92], while the vaspin-kallikrein 7 complex has 70 kDa [90]. Its expression was noted for the first time in the visceral adipose tissue (VAT) of rats [89], and then in many tissues, including the liver, pancreas [94], skin [95], placenta [96], stomach, cerebrospinal fluid, hypothalamus [97], and ovaries [98]. Its levels in plasma and follicular fluid were noted to be around 0.18 to 1.55 ng/mL in humans [99], and at the level of 1 ng/mL in pigs [98]. The literature indicated several vaspin expression regulators. For example, its level depends mostly on body weight; significantly lower concentrations of the adipokine were observed in the serum of underweight children [100], and the mRNA of vaspin in the adipose tissue was increased by body mass increase [101]. Besides, other factors increasing vaspin expression in VAT and serum are insulin, insulin resistance, and leptin elevation, while in liver, exposure to a high-fat diet was described in many species, including rats, mice, and humans [102]. Moreover, in porcine ovarian follicles, vaspin expression was upregulated with fattening [98]; all these findings clearly indicate its compensatory role in obesity.

The 78 kDa glucose-regulated protein (GRP78), also named as heat shock protein family A member 5 (HSPA5) [103], was described as a vaspin receptor. The GRP78 is encoded by a gene found in humans on chromosome 9. The HSPA5/GRP78 consists of 4532 nucleotides located in eight exons [104]. The functional gene promoter is divided into two parts, the distal domain elevating the basal expression of GRP78 and the proximal regulatory response to various stimuli [104]. The encoded protein with a molecular weight of 78 kDa is built from 654 aa in humans [105]. The GRP78 structure consists of three domains: 10 kDa *C*-terminal tail, and 20 kDa domain C-terminus which binds polypeptides, and 44 kDa domain in N-terminus binding ATP [106]. The receptor gene expression was detected in the brain, thyroid, thymus, and adipose tissue, as well as the placenta, ovary, and testes of humans, rats, and mice [105]. Protein abundance was noted mostly in the endoplasmatic reticulum (ER) lumen, and its levels were elevated under the influence of ER stressors, such as sugar deficit, and inhibited protein glycosylation, or in disturbing intracellular calcium storage [104]. The GRP78 expression is regulated by different hormones, for example, in cows’ ovaries, its mRNA was elevated by FSH (follicle-stimulating hormone) [107], whereas in rats, it was elevated by PGF_2α_ [108]. Furthermore, in human neuronal cells, leptin stimulates GRP78 protein [109]. Interestingly, the receptor expression depends on the fattening level in the porcine adipose tissue [110], while in mice, liver was inhibited by caloric restriction [111]. The GRP78 mainly regulates peptide translocation throughout the ER membrane targets misfolded proteins to degradation in ER, as well as regulates cell survival [112]. Interestingly, in the reproductive tract, GRP78 regulates CL function maintenance via the inhibition of caspase activation, affecting the capacitation of sperm and participating in uterine receptivity/sensitization, as well as embryo development [113].

Vaspin binding to GRP78 may regulate the physiology of different cell types. The probable mechanism depends on GRP78 [103] and vaspin [114] affinities for negatively charged cell membrane phospholipids. Moreover, binding sites in the GRP78 hydrophobic region have not been described yet, while vaspin binds GRP78 via helical domains in the N-terminus [103]. Vaspin activates multiple kinase pathways which allows for pleiotropic function in the organism. For example, AKT pathway activation stimulates insulin secretion in rats’ pancreatic islets [115], as well as relating to the osteogenic differentiation in mice [116]. Furthermore, AMPK phosphorylation and nuclear factor kappa B subunit 2 (NFKB2) downregulation inhibit the level of gene expression of adhesion molecules in human vascular endothelial cells [117]. Through the phosphorylation of MAPK/p38, vaspin inhibits apoptosis in human osteoblasts [118]. Moreover, by activation of GRP78 and PKA, but not MAP3/1 pathways vaspin stimulates ovarian follicular steroidogenesis [119], while, via the activation of MAP3/1, AKT, and STAT3, participates in proliferation induction and apoptosis inhibition in pigs [120]. Interestingly, via MAP3/1 and PRKAA1, vaspin also regulates in vitro porcine oocytes maturation [121].

### 3.6. Chemerin

Chemerin, another member of the adipokines group, was firstly described by two independent research teams in 2003 as the natural ligand of chemokine-like receptor 1 (CMKLR1, ChemR23), an orphan GPCR related to the chemokine receptors [122,123]. The hormone, known also as retinoic acid receptor responder protein 2 (*RARRES2*) or tazarotene-induced gene 2 (TIG2) protein, was identified as a product of the gene with the expression up-regulated under the influence of tazarotene, the RAR β/γ-selective anti-psoriatic agent [124]. The *RARRES2* gene consists of six exons and five introns and, in humans, is located on chromosome 7q36.1. The coding sequence of the porcine chemerin gene shared about 87.7% and 72.3% similarities with human and mouse sequences, respectively [125]. The human chemerin aa sequence shares about 66%, 63%, 76%, and 84% identities with rat, mouse, cattle, and pig sequences, respectively [126]. *RARRES2* encodes a biologically inactive hormone precursor called prochemerin. In humans, prochemerin consists of 163 aa with a 20-aa hydrophobic signal peptide [127]. In the bloodstream, several different extracellular serine-, cysteine- or carboxypeptidases cleave prochemerin C-terminus, which results in the formation of active hormone isoforms. The diversified activity of the hormone depends on its cleavage site and the type of involved protease [126].

Chemerin has been found to exert pleiotropic effects, including the modulation of insulin sensitivity and the regulation of food intake, energy homeostasis, and adipose tissue function [128,129]. The hormone has also been found to exert opposite, pro- and anti-inflammatory effects in the organism. Chemerin was reported to act as a chemotactic agent for immune cells such as leukocytes, macrophages, and immature dendritic cells during inflammation [128]. However, it has been indicated that adipokine inhibits the secretion of pro-inflammatory cytokines, such as interleukin-6 (IL-6) and tumor necrosis factor-α (TNFα) [128]. The expression of chemerin was confirmed in different tissues of various species, such as mice, rats, cattle, pigs, and poultry [49,125,130,131,132]. In humans, besides the WAT, the expression of the hormone was confirmed in the brown adipose tissue, liver, lungs, kidneys, skeletal muscles, ovaries, and placenta [133,134,135]. In human blood, the hormone concentration reached levels of 100 to 200 ng/mL [136]. Plasma chemerin concentration was found to be correlated with body mass index (BMI), as well as with age and sex [134]. In pigs, the plasma chemerin concentration was determined at the level of 70 to 160 ng/mL, and was dependent on the animals’ physiological status (the period of the estrous cycle or stage of pregnancy) [137].

Chemerin exerts its influence through binding to three GPCRs: CMKLR1, G protein-coupled receptor 1 (GPR1), and C-C motif chemokine receptor-like 2 (CCRL2). CMKLR1 is the best-known chemerin receptor. GPR1 has a similar structure to CMKLR1, however, its role has not been fully explored. Due to the fact that both receptors are expressed in different tissues, it is assumed that they may play different functions. The presence of CMKLR1 has been confirmed mainly in cells connected with the immune system, such as macrophages, natural killer cells, plasmacytoid dendritic cells, and myeloid dendritic cells, while the expression of GPR1 was detected mostly in cells related to the central nervous system (CNS) [123,138,139]. Variable levels of chemerin expression were observed in the porcine hypothalamus during the estrous cycle; higher in the early- and mid-luteal phases [137]. Chemerin and CMKLR1 were also detected at the level of mRNA and protein in mouse and human ovary under the physiological state [133]. The signal transduction mechanism of the chemerin receptors is based on MAPK/ERK1/2 and PI3K/AKT pathways (through CMKLR1), and on the AMPK signaling pathway (through both CMKLR1 and GPR1) [140]. Moreover, it was indicated that chemerin, by binding to CMKLR1, may initiate the influx of intracellular Ca^2+^, the repression of cAMP, and the phosphorylation of p42–p44 MAP kinases [123]. The structure of the third chemerin receptor, CCRL2, prevents it from the transduction of signal into the cell. However, it was indicated that the receptor binds the *N*-terminal region of the adipokine and exposes chemerin’s C-terminus to CMKLR1 localized on the other cells [141]. The expression of CCRL2 was reported, i.a., in the immune system cells, such as T cells, macrophages, and neutrophils [139].

In general, chemerin has an inhibitory effect on ovarian steroidogenesis. In human Gc, it decreases both basal and IGF-1-induced P_4_ and E_2_ secretion [142]. The inhibition of in vitro oocyte maturation in cattle through MAPK/ERK1/2 kinases pathways under the influence of the hormone was also noted [140].

### 3.7. Orexins

Orexin A (OXA) and B (OXB) are hypothalamic-derived neuropeptides that arise as products of proteolytic cleavage from a common 130 aa precursor called prepro-orexin (PPO) [143]. The human *PPO* gene is located on chromosome 17q21 and consists of two exons and one intron, 1432 bp in length. The first exon, consisting of 143 bp, includes the 5′-untranslated region and a small part of the coding region. The coding region delivers the sequence of the first seven residues of the secretory signal sequence. The second exon consists of the sequence encoding the rest of the open reading frame, as well as the 3′-untranslated region [144,145]. The porcine *PPO* gene is located on the SSC12 region of chromosome 12 and consists of one intron and two exons with an overall length of 1247 bp [146]. As mentioned above, both hormones are products of the proteolytic cleavage of 130 aa prepro-orexin protein. The estimated aa sequence homology between OXA and OXB is 46%. OXA, considered the more biologically active one, contains 33 aa and has a molecular weight of 3.5 kDa. OXB has a molecular weight of 2.9 kDa and contains 28 aa [144,145]. The hormones were originally discovered in the rat lateral and posterior hypothalamus, structures involved mainly in the control of energy homeostasis and food intake [144,145]. Despite the fact that orexin-positive cell nuclei were localized mainly in the lateral and perifornical areas of the hypothalamus, their fibers project through all structures of the CNS [147,148]. The neuropeptides were also identified as modulators of sleep regulation and arousal [149]. Orexins have also been found to play a role in the rewarding process and addiction, sensory modulation, stress processing, locomotion, and cognition [150,151,152,153,154]. The concentrations of orexins were determined in the plasma of different species. In rats, OXA concentrations reached the level of 12 to 14 pg/mL [155]. In humans, the concentrations of OXA and OXB were determined at the levels of 6.76 to 1000 pg/mL and 670 pg/mL, respectively [156,157]. In pigs, during the estrous cycle, the serum OXA level varied from 210 and 350 pg/mL, whereas OXB—from 210 to 380 pg/mL [158]. During the early gestation period, porcine OXA and OXB concentrations were determined at 102 to 704 pg/mL and 40 to 4077 pg/mL, respectively. A growing body of evidence indicates the role of orexins in the regulation of different endocrine axes, including the HPO axis [159,160,161,162,163,164,165].

Orexins have a pleiotropic effect through binging to two GPCRs, containing seven transmembrane domains: orexin receptor type 1 (OX1R) and type 2 (OX2R). While OX1R was found to be highly selective for OXA, OX2R binds both orexins with similar affinity [144,145]. These receptors share 94% and 95% sequence identity for both humans and rats, which indicates the high conservativeness between the species. Both receptors have been found to mediate the effect of orexin binding through activating PKC, as well as elevating the intracellular Ca^2+^ ions level [166]. Several research works indicate that orexins may act on the target cells through a number of signaling pathways, including PI3K, ERK 1/2, p38, AKT, and MAPK [167,168,169,170,171,172,173]. For more, it was also indicated that orexins may exert their effect by affecting cAMP synthesis and via the activation of the mTORC1 signaling pathway [174,175,176]. In the central nervous system, orexin receptors have been localized in many brain regions, including the hippocampus, amygdala, anterior and lateral hypothalamus [177,178,179]. Besides the central nervous system, the orexin receptors are widely expressed in a variety of species and tissues. OX1R and OX2R were localized in the human WAT, digestive tract, and pancreas, as well as in testes, endometrium, and placenta [180,181,182,183]. In rat, the expression of the receptors was confirmed, i.a., in the pituitary, thyroid, jejunum, gonads, lungs, and kidney [155]. In pigs, both orexins have been found to be expressed in the adipose tissue, pancreas, pituitary, ovaries, uterus, trophoblasts, and embryos [161,162,180,184,185,186]. The relationship between orexins and gonadal steroids, of which the production is regulated by orexins, is reciprocal, and does not just take place at the ovarian level. Plasma concentrations of OXA and OXB fluctuated during the estrous cycle in pig, which strongly suggests their dependence on gonadal steroids’ action [158]. Similarly, orexins levels and the expression of their receptors in the hypothalamus and pituitary in rats [187,188,189,190,191] and pigs [161,192] were dependent on animal hormonal status related to the phases of the estrous cycle, pregnancy, gender, the administration of steroid hormones, and gonadectomy.

The occurrence of orexin receptors in the hypothalamus and pituitary, two higher branches of the HPO axis, allows the indirect effect of orexins on the reproductive system by controlling the secretory activities of both structures. Orexin A enhanced GnRH release in rat hypothalamic explants harvested from females at proestrus [193]. Orexin A also induces *GnRH* gene expression and release from GT1-7 neurons [194]. On the other hand, it has been shown that orexins injected into the third ventricle inhibited LH secretion in ovariectomized rats [195]. In the case of the pituitary, there was found a stimulatory effect of both orexins on basal FSH and LH secretion by anterior pituitary cells of proestrus rats [196], and inhibitory influence of OXA on GnRH-induced LH release by these cells [193]. Immunohistochemical analysis indicated the presence of the orexin system in porcine ovarian follicles. Moreover, OXA affected the steroidogenesis and proliferation of Gc [197]. In early pregnant pigs, the variable and regulated by P_4_ expression of PPO, OX1R, and OX2R was noted in the endometrium, trophoblast, and embryo [186,198].

### 3.8. Ghrelin

Ghrelin was firstly identified in 1999 in the rat stomach as a gut-derived ligand of an orphan GPC receptor, which is able to stimulate the secretion of growth hormone (GH) [199]. The hormone is known for its pleiotropic effects on the organism. In humans, the ghrelin gene called ghrelin and obestatin prepropeptide (*GHRL*) is composed of four coding exons and a short first exon of 20 bp, which is termed exon 0. Exons from 1 to 4 encode a 117 aa precursor called prepro-ghrelin. Exon 1 encodes the signal peptide, whereas the 28 aa coding sequence of the hormone is encoded by parts of exons 1 and 2. Exon 3 of the gene was found to encode another hormone, obestatin [200,201,202]. Human, mouse, and rat *GHRL* genes were mapped to chromosomes 3p26-256, E3|6 52.84 cM, and 4q42, respectively [202,203]. As mentioned above, prepro-ghrelin is a precursor of two peptides: ghrelin and obestatin. Ghrelin is a 28 aa peptide, with the n-octanoylated modification on the serine 3. The modification was found to be essential for the hormone’s biological activity [199]. Later, studies on the structure of the hormone revealed another possible modification of its sequence involving the deletion of Gln14 [204]. The expression of the hormone gene and/or protein has been confirmed in many species and tissues. The highest amount of ghrelin’s encoding mRNA was observed in the gastrointestinal tract and pituitary, whereas the lowest was in the lungs and ovaries [205]. The expression of the hormone was also confirmed in the CNS, gastrointestinal tract, and reproductive tract of rats and pigs, as well as in the CNS and gastrointestinal tract of mice [199,206,207,208,209,210,211,212,213,214]. In humans, there were normal plasma ghrelin concentration estimates on 10 to 20 fmol/mL for n-octanoyl modified hormone and 100 to 150 fmol/mL for total ghrelin, including acyl-modified and des-acyl form of ghrelin. The plasma hormone level has been found to be elevated during fasting, and reduced in obese individuals [215,216]. In rats, the plasma levels for n-octanoyl form and total ghrelin concentrations were at the levels of 4.02 fmol/mL and 219.6 fmol/mL, respectively [217]. In pigs, the plasma level of the total hormone was reported to be at a level of 67.2 pg/mL [218]. Ghrelin may take part in the regulation of metabolism affecting food intake and body weight [219]. The hormone is also involved in the regulation of processes such as GH release, cardiovascular system functioning, and gastrointestinal motility [220,221,222]. Furthermore, the hormone has also been shown to be involved in the mechanisms of learning and memory, reward/addiction, and depression/anxiety [223,224,225,226].

The presence of the receptor responsible for the stimulation of GH release in the pituitary and hypothalamus of pigs was firstly described by Howard et al. [227] in 1996, and it was called GH secretagogue receptor (GHS-R). GHS-R belongs to the GPCRs family and consists of, depending on the species, from 364 to 367 aa, and seven transmembrane domains [227,228,229]. Two GHS-R subtypes were identified; the full-length type 1a receptor and the truncated type 1b. The hormone receptor subtypes are the effect of alternative splicing of a single gene. Since GHS-R1a is the functionally active, signal-transducing form of the receptor, GHS-R1b lacks two domains, 6 and 7, and is unable to bind a ligand and transduce a signal [229]. Due to the negligible role of GHS-R1b in the ghrelin actions, herein we will focus only on GHS-R1a. The GHS-R1a structure is characterized by a high homology between species. Human and rat polypeptide chain sequences share 96.1% homology, whereas human and porcine sequences share a 94.5% similarity [229]. Furthermore, the transmembrane region of the receptor is even more conservative, showing over 98% sequence similarity in these species [230]. The mechanism of ghrelin signal transduction is based primarily on the changes in Ca^2+^ ions concentration via the activation of G-protein subtype Gaq/11. The activation of the Gaq/11 subtype results in the activation of phospholipase C (PLC) and PKC, the production of inositol triphosphate (IP3), and, as a result, the release of Ca^2+^ ions [231]. Ghrelin has also been found to mediate its effect via the activation of the ERK1/2, PI3K, and AKT kinases, and the activation of the AMPK signaling pathway [232,233,234,235,236]. GHS-R1 is widely expressed in mammalian tissues. In the CNS, the expression of the receptor has been reported in the hypothalamus, and pituitary of rats, humans, and pigs [227,237,238]. Besides the CNS, the expression of GHS-R1a was also confirmed in many other peripheral tissues. In humans, the expression of the receptor was confirmed in the adrenals, myocardium, thyroid, spleen, and pancreas, as well as in testis, ovaries, and endometrium [239,240,241]. In rats, the expression of ghrelin receptor has also been localized in gastrointestinal tissues, kidneys, pancreas, and testes [217,242,243,244,245]. In pigs, besides CNS, the receptor has been reported in ovaries and testes [246,247].

In general, ghrelin influences the reproductive system by inhibiting LH secretion in humans, rats, sheep, and monkeys [248]. Similarly, at the ovary level, ghrelin downregulated the secretion of steroids: testosterone (T), E_2,_ and P_4_, which is also seen in the altered expression of HSD3B, hydroxysteroid 17-beta dehydrogenase 1 (HSD17B1), and cytochrome P450 family 19 subfamily A member 1 (CYP19A1/P450_AROM_) proteins in mature pigs [249]. Interestingly, in the in vitro culture of the ovarian cells from prepubertal pigs, ghrelin exerts the opposite effect on steroidogenesis [250]. The expression of ghrelin and *GHS-R1a* mRNAs has also been shown in chicken ovary, where ghrelin is able to induce proliferation markers and, at the same time, reduce apoptosis markers and stimulate the secretion of P_4_, E_2_, arginine-vasotocin, and IGF1 [251]. Ghrelin can also influence the oocyte maturation with a negative effect on cumulus cells viability in cattle [252]. Elevated serum ghrelin levels are observed in women suffering from PCOS [253].

### 3.9. Kisspeptin

In 1996, Lee et al. [254] isolated, for the first time, Kiss1 from the melanoma cell line. The kisspeptin gene was officially named *KISS1* [254]. In 2001, Kotani et al. [255] found that Kiss1 was a natural ligand of GPR54, previously considered an orphan GPCR. Thereby, GPR54 was also called Kiss1R [255]. The receptor and its ligand are collectively called the Kiss1 system. In humans, *KISS1* is translated to preprokisspeptin, including the signal peptide to be loaded to the transporting vesicles. Next, the peptide is proteolytically cleaved at the next site to the dibasic residues by the subtilisin-like convertase. The *C*-terminal of nuclear receptor coactivator 4 (RFG) is amidated by the carboxypeptidase. The human Kiss1 is composed of 145 aa propeptide cleaved into a 54 aa peptide, which may be processed to a shorter peptide of 10, 13, or 14 aa. The kisspeptin protein sequence is highly conserved between species [256]. Kisspeptin-54, kisspeptin-14, and kisspeptin-13 were purified from human placenta [58,257]. In cattle, sheep, and goats, the longest form is kisspeptin-53, and in mice, it is kisspeptin-52 [258].

In 2013, Bianco and Kaiser predicted the KISS1R structure in cellular membrane considering the aa sequence, which results in three extracellular and three intracellular loops and seven transmembrane helices [259]. The intracytoplasmic *C*-terminal region binds to the regulatory and catalytic subunits of phosphatase A2 [260], and then the Kiss1R signal induces the increase of Ca^2+^ levels, the activation of calcium-dependent signaling pathways, MAPKp38, and ERK1/2 kinases in GnRH neurons. Indeed, when Kiss1 binds its receptor, there is an activation of the Gq/11-mediated PLC signaling pathway, and then a release of intracellular Ca^2+^ and arachidonic acid [255]. It is suggested that this signaling cascade allows GnRH release via the GnRH neurons depolarization [261].

In mammals, the Kiss1 system has an important role in reproduction [262], but also a potential role in metabolism [258]. In 2003, the Kiss1 role in reproduction was first discovered in human patients. The loss of function due to the mutation of GPR54 leads to hypogonadotropic hypogonadism characterized by a deficiency in LH and FSH secretion, infertility, and a lack of puberty onset [263,264]. The same results were observed with transgenic mice which did not express Kiss1 or its receptor [263]. Kisspeptin expression is influenced by leptin. Indeed, leptin receptors were found in Kiss1 neurons in the arcuate nucleus of ob/ob mice [265]. In humans, the Kiss1 system is expressed in spermatozoa [266], and Kiss1 is also expressed in the ovarian follicle, but it is stronger in cumulus cells than in mural Gc [267].

### 3.10. Phoenixin

In 2013, with the use of modern bioinformatics tools, advanced algorithms, and databases, such as the Human Genome Project, the Samson group identified a hitherto unknown peptide, which was called phoenixin. Successively, it was possible to confirm its presence in many species, including humans, rodents, pigs, cows, or zebrafish [268]. Phoenixin is produced mainly in the hypothalamus by the proteolytic cleavage of a small integral membrane protein 20 (*SMIM20*). In humans, a gene for this precursor is located on chromosome 4 at position p15.2 [269]. The hormone exists in many isoforms which differ in the length of aa sequences and are named phoenixin-42, -36, -26, -20, -17 and two predominant forms—phoenixin-20 and -14, which are the most active, and occur in larger amounts in many tissues. It is a highly conserved peptide among species, for instance, phoenixin-14 has identical sequences in humans, rats, mice, and pigs, while phoenixin-20 differs in one amino acid between the coding regions of human, canine, and porcine sequences [268]. The highest expression of this peptide was noted in the rat hypothalamus. Additionally, in particular, the co-expression of phoenixin-14 and nesfatin-1 occurs at a high range of 70–86% [270]. This peptide is also widely expressed in peripheral tissues, beginning from the heart [271], thymus, stomach [268], pancreas [272], lung, and kidney [273], and in the adipose tissue [274], and ovary [275,276]

In 2016, Stein et al. using the deductive receptor-matching strategy, and proposed the G protein-coupled receptor 173 (GPR173) as a candidate for the phoenixin receptor. So far, the studies on the influence of phoenixin seem to confirm this assumption. The use of GPR173 siRNA abolishes the action of phoenixin in the brain, heart [277], and ovarian follicles [276]. The GPR173 also termed in literature as SREB3 belongs to the superfamily named the super conserved receptor expressed in the brain (SREB) and consists of seven transmembrane helical domains, each with one site of phosphorylation [278]. In the case of this receptor, phosphorylation causes its desensitization and endocytosis [279]. Interestingly, similar asparagine sites at the N-terminus have receptors for GnRH and LH, suggesting the importance of GPR173 in the regulation of reproductive functions [280,281]. Until now, no endogenous agonists have been found for GPR173, but some evidence indicates that the GnRH-derived peptide formed after cutting this hormone by endopeptidase may bind to GPR173 to inhibit nerve cell migration in wound healing [282]. It is plausible that some effects of phoenixin can be GnRH receptor-dependent, e.g., cetrorelix, GnRH receptor (GnRH-R) antagonist, abolishing phoenixin-induced memory recognition and anxiolytic effects [283]. Phoenixin-20 can increase the level of cAMP as well as the phosphorylation of ERK1/2 and CREB (cAMP response element-binding protein), indirectly activating PKA to stimulate the expression of GnRH mRNA [284].

The existing literature indicates the involvement of phoenixin in metabolism, e.g., the development of obesity, insulin resistance, or the pathogenesis of inflammatory reactions of the body, and increased food intake. The effects of this neuropeptide improve memory and reduce anxiety [277,285]. In humans, the association of phoenixin with long-term changes in body weight has been described in PCOS patients where a positive correlation between BMI and phoenixin levels has been demonstrated [286]. Billert et al. showed that phoenixin-14 is involved in the proliferation and differentiation of 3T3-L1 cells and primary rat preadipocytes, thus promoting adipogenesis [274].

Studies on the role of phoenixin in female reproduction are still limited. Nevertheless, it is well established that this neuropeptide acts on higher branches of the HPG axis. Indeed, the intracerebroventricular injection of phoenixin-14 increases GnRH levels in female rats [287]. In zebrafish, phoenixin 20 can also increase the expression of GnRH-R and kisspeptin [288]. It is notable that both the GnRH-R agonist (buserelin) and antagonist (cetrorelix) can modulate the expression of *SMIM20* and *GPR173* in the entire HPO axis in adult rats [289]. An in vivo study indicated that the injection of both active isoforms of phoenixin significantly increased LH plasma levels in female rats [268]. Conversely, the study conducted in fish *Scatophagus argus* showed that phoenixin stimulates the gene expression of gonadotropins, LH and FSH, as well as the GnRH-R in the pituitary, without affecting GnRH in the hypothalamus, indicating that phoenixin in fish can regulate the HPO axis directly at the pituitary level [290]. The latest study of Nguyen et al. showed phoenixin and GPR173 expression in women’s ovaries, and its beneficial effect on ovarian steroidogenesis and Gc proliferation [276]. In recent years, the first works on the involvement of phoenixin in pathologies of the reproductive system such as PCOS appeared. A higher phoenixin-14 level was observed in women with PCOS compared to the controls, and it was positively correlated with LH, FSH, total T level, and BMI [286]. Our team’s research also indicates a higher phoenixin-14 level in the plasma of rat model of PCOS; we observed a higher *SMIM20* mRNA expression in the ovary and adipose tissue, while phoenixin-14 peptide production was higher only in the ovary of the PCOS rat [275].

## 4. Expression and Function of Adipokines and Neuropeptides in the Corpus Luteum

### 4.1. Leptin

Although most studies focus on the presence of leptin and its receptors in Tc and Gc, as well as on the role of the adipokine in the regulation of ovarian follicles and oocytes physiology, there is evidence for leptin system expression also in the CL of various species. The presence of leptin mRNA and protein has been described in the CL of humans [291], rats [292], pregnant and non-pregnant pigs [293,294,295], cattle [296], mares [297], goats [298], and water buffalo (*Bubalus bubalis*) [299] (Table 1). The leptin transcript has also been found in canine CL during pregnancy and the estrous cycle [300], and the adipokine protein has been immunolocalized in the murine LCs [301]. The expression of LEPR transcript and protein has been reported in the CL of rats [301,302], pregnant and non-pregnant pigs [294,303,304,305,306], mares [297], and water buffalo [299]. Leptin receptor mRNA presence has also been observed in the CL of humans [291], pregnant baboons [307], pregnant and non-pregnant cattle [296,308], and bitches [300]. Additionally, the expression of the receptor protein has been reported in murine [309], rabbit [310], alpaca [311], and Japanese black bear [312] CL. The differentiated expression of the leptin system components dependent on the stage of CL functioning suggests that it may be affected by steroid hormones [292,293,296,297,299,300,306,308]. The in vitro studies of isolated porcine LCs revealed an up-regulatory role of E_2_, P_4_, and LH on leptin transcript content and the protein secretion [313,314]. Moreover, the study of Ryan et al. showed that human chorionic gonadotropin (hCG) may also increase the mRNA level of the adipokine and both variants of the leptin receptor, LEPRa, and LEPRb, in the murine CL [309].

**Figure 2:**
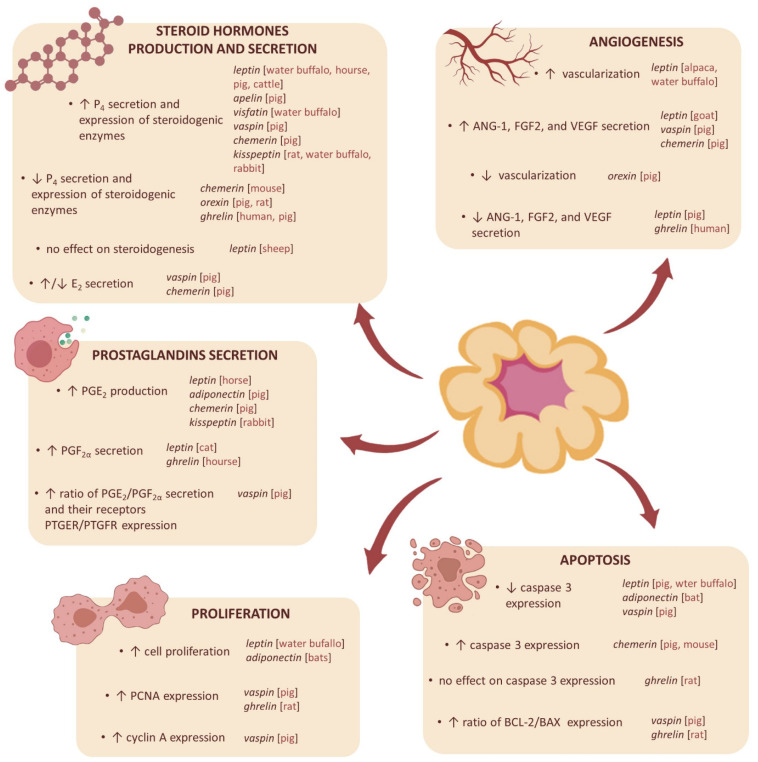
The influence of adipokines and neuropeptides on the functions of the corpus luteum in humans and various animal species. P_4_—progesterone, E_2_—estradiol, PGE_2_—prostaglandin E_2_, PGF_2α_—prostaglandin 2α, PTGER—prostaglandin E receptor, PTGFR—prostaglandin F re-ceptor, PCNA—proliferating cell nuclear antigen, VEGF—vascular endothelial growth factor, FGF2—fibroblast growth factor 2, ANG-1—angiopoietin 1, BCL-2—B-cell lymphoma-2 protein, BAX—Bcl-2-associated X protein, ↑—increase, ↓—decrease.

In pigs, during the early luteal phase, leptin added alone and in combination with GH had no effect on P_4_ secretion by luteal cells [315]. In this period, leptin decreased caspase 3 activity in the porcine CL (Figure 2). On the other hand, treatment with leptin and IGF1 suppressed P_4_ secretion and stimulated the apoptosis rate. In mature CL, leptin added concomitantly with IGF1, diminished P_4_ production, however, no effect on caspase 3 activity was found. It has been concluded that the action of leptin is restricted to the stage of CL formation. During the early luteal phase, leptin acts as an anti-apoptotic factor, which is necessary for the maintenance of homeostasis in developing CL [315]. In water buffalo, leptin alone exerted a significant stimulatory effect on P_4_ synthesis in a dose- and time-dependent manner; however, in the presence of IGF1, an antagonistic effect was observed [316]; it has been found that there was a gradual increase in the expression profiles of the genes and proteins responsible for steroidogenesis, such as STAR, CYP11A1, HSD3B1, with respect to dose and time duration, and this contributed to increased P_4_ synthesis. Moreover, leptin had an inhibitory effect on apoptosis promoting cellular proliferation and angiogenesis [316]. These observations suggest that, in water buffaloes, leptin regulates ovarian steroidogenesis, angiogenesis, and cell proliferation, and it can control the effect of systemic factors, such as IGF1 [316]. Moreover, a direct stimulatory, dose-dependent effect of leptin on P_4_ secretion by CL was reported by Galvão et al. [297] in equine during the early- and mid-luteal phases of the estrous cycle. Similarly, in the bovine CL during the early luteal phase, leptin, at physiological concentrations (10 ng/mL), in the presence of IGF-1 (100 ng/mL), caused a significant increase in P_4_ secretion by LCs [308]. These results suggest that leptin may also be the regulator of steroidogenesis in horses and cattle. In ewes, ovarian exposure to physiological concentrations of leptin on day 3 of the estrous cycle did not affect P_4_, E_2_, or LH concentrations compared to control [317]. These results suggest a lack of leptin effect on ovarian functions at this stage of the cycle in this species, and that the effect of leptin on steroidogenesis may be species-specific. Furthermore, E_2_ and P_4_ increased leptin gene expression and leptin secretion by the porcine LCs, indicating that steroid hormones affect leptin mRNA levels and leptin secretion during the mid-luteal phase of the estrous cycle and early pregnancy [313,314]. Thus, it seems that not only does leptin affect steroid hormones secretion, but steroids can also affect leptin expression.

Studies on the influence of leptin on prostaglandin secretion by the ovary are limited to two experiments. In the equine early luteal cells, leptin increased PGE_2_ secretion in a dose-dependent manner [297]. In cats during proestrus, leptin promoted PGF_2α_ release by the cultured ovarian fragments [318]. Growing evidence indicates that leptin can stimulate blood vessel growth and CL development. In vivo studies in alpacas have shown that the administration of leptin during pre-ovulatory fasting increased the vascularization of the CL, and a positive correlation between vascularization, CL diameter, and plasma P_4_ levels was demonstrated [319]. Induced leptin deficiency (after anti-leptin antibody treatment) during the growth and maturation of the caprine CL resulted in an increase in the number of large diameter vessels, the number of undeveloped CLs with abnormal morphology, and a higher ratio of LLCs to SLCs. Leptin replacement therapy following an induced leptin deficiency promoted normal tissue development, an increase in overall tissue mass, and the formation of a structure that resembled the mature CL [298]. The results of in vitro studies seem to confirm the role of leptin in the formation of CL. In goats, leptin stimulated *ANG-1*, *FGF2*, and *VEGF* gene expression, but only in early-stage luteal cultures [320]. On the other hand, in the cultured porcine LCs, isolated from days 5–7 of the cycle, leptin dose-dependently decreased *VEGF*, *ANG-1*, and *FGF2* mRNA abundance [321].

### 4.2. Adiponectin

Chabrolle et al. [54] found that the adiponectin system, including adiponectin and its two receptors AdipoR1 and AdipoR2, was highly expressed in adult rats CL; it was the first time that AdipoR1 and AdipoR2 were characterized in the rat ovary. Immature females were treated by pregnant mare serum gonadotropin (PMSG) for 24h, and then with hCG for 48 h. Using immunohistochemistry, they found that the adiponectin system was more expressed in CL than in Gc. PMSG and hCG induce ovulation and luteinization, so the authors hypothesize that an increase in adiponectin and AdipoR1 protein could be due to a high proportion of CL in the ovary in response to hCG treatment, suggesting that adiponectin could be associated with rat luteal growth and development [54]. Maillard et al. discovered, for the first time, that adiponectin, AdipoR1, and AdipoR2 were expressed in bovine CL [322]. Tabandeh et al. [323] studied the expression of adiponectin and its two receptors AdipoR1 and AdipoR2, along with the active CL lifespan and in regression in the bovine ovary. They found that, at the beginning of CL growth, there is a decrease of adiponectin system expression, while during CL regression, the adiponectin system is more expressed [323]. Campos et al. [324] found that adiponectin, AdipoR1, and AdipoR2 are expressed in the porcine CL; they classified 2 groups of hypo-fertile and hyper-fertile gilts based on the number of piglets of the two previous gestations. They discovered that sub-fertile sows have lower levels of circulating adiponectin in plasma and in follicular fluid, which correlate with the inhibition of steroidogenesis and lower number of CL, while in adipose tissue, adiponectin expression is higher. Additionally, it also notes the alerted expression of adiponectin system in the ovary: the protein content of adiponectin is lower in the CL of sub-fertile pig; in contrast, AdipoR1 and AdipoR2 levels increase [324]. In pigs, adiponectin mRNA expression is higher in CL than in Gc and Tc during days 2 to 3, 10 to 12, and 14 to 16 of the luteal phase. For the protein, adiponectin is less expressed at days 10 to 12 of the luteal phase [325]. In buffalo, the adiponectin system is expressed at each stage of the luteal phase, but it varies depending on the stage. The adiponectin system is more expressed during the early luteal stage and regression [326].

Maleszka et al. [325] showed that porcine LCs in vitro exposed to physiological concentrations of adiponectin have shown a decrease of P_4_ concentration only in cells derived from CL in the middle of the luteal phase, but not at the beginning and the end of the luteal phase. Recombinant adiponectin induces the gene expression of prostaglandin E synthase (*PGES*) in porcine Gc [327], which is a key limiting enzyme in the prostaglandin biosynthesis pathway [328]. Furthermore, recombinant adiponectin induces the gene expression of cyclooxygenase-2 (COX2) [327]. Sakurai et al. [329] discovered that COX2 activity could be linked to functional CL because of its capacity to stimulate angiogenesis in immature rats. Few data are available about the impact of adiponectin on proliferation and apoptosis in CL. However, Anuradha et al. [330] hypothesized that the high concentration of adiponectin in CL could allow the prevention of apoptosis in LCs. When bats were treated with adiponectin during late embryonic development, there was an increase in cell proliferation markers, such as proliferating cell nuclear antigen (PCNA), and a decrease in active caspase 3, thus, adiponectin could allow the reactivation of luteal activity, and then could prevent the apoptosis of LCs [330].

### 4.3. Apelin

The expression of apelin was confirmed in the CL of different species, for example, Shirasuna et al. [331] demonstrated, for the first time, the presence of apelin and APJ in the bovine CL. The authors show that the mRNA expression of apelin and *APJ* increases through the luteal phase with the highest peak in the late stage of CL for apelin and the regression luteal phase for *APJ*. Shirasuna et al. [331] also detect the mRNA and protein expression of both apelin, and APJ only the smooth muscle cells of luteal arterioles. Additionally, Shilffarth et al. [332] obtained similar results; the authors showed that apelin mRNA levels increase in early and mid-luteal phases in the bovine ovary. The protein expression of apelin and APJ was also confirmed in adult ewes; the authors of this study demonstrated that the expression of apelin and APJ mainly occurs in the LLCs [333]. Xu and Stouffer in 2012 [334] reported that apelin and APJ are expressed in the CL of rhesus monkeys. The authors demonstrated high levels of apelin in the CL in the early- to mid-late luteal phases, with a decline in the late luteal stages. Differences in APJ expression have also been demonstrated by the authors: low levels in the early luteal phase and the highest levels in the mid–late luteal phase, and a decline in expression in the very late luteal phase [334]. The expression of apelin and APJ (mRNA and protein) has also been confirmed in the CL of pigs [67]; the quantity of apelin (mRNA and protein) was similar in early and mid-luteal CL and then decreased in regressing CL and APJ amount is the highest in mid-luteal CL. Różycka et al. [67] also demonstrated differences in the immunolocalization of apelin in SLCs and LLCs during the early, mid- (the highest intensity), and late luteal phases. Additionally, Pirino et al. [335] showed the protein expression of the apelin system in the CL (in the cytoplasm of some LCs) from dogs’ ovaries, and Pope et al. [65] demonstrated the expression of apelin and APJ in mice ovaries, mainly in the periphery of the corpora lutea mass.

Literature data about the effect of apelin on CL physiology are limited. Available studies show that apelin plays a crucial role in CL luteolysis, angiogenesis, and steroidogenesis. For example, Shirasuna et al. [331] reported that after 0.5–2 h incubation with PGF_2α_, the mRNA expression of apelin and *APJ* increased in the bovine CL, but after 4 h incubation with this compound, the mRNA expression of the apelin system was decreased, when compared to the control. These results suggest that PGF2 α, at the early stage of luteolysis, stimulates the expression of apelin and APJ, which may be a local regulator in the bovine CL involved in luteal blood flow [331]. Additionally, apelin has an impact on the steroid hormones secretion; Różycka et al. show that apelin stimulates P_4_ secretion in the porcine CL via the activation of AMPK signaling pathway and the modulation of HSD3B1 expression [67].

### 4.4. Visfatin

The expression of visfatin in the CL of water buffalo is best documented. In this species, the abundance of both visfatin transcript and protein is the highest in the late CL compared to the CL from other luteal stages, while interestingly, in regression CL, it is the lowest. Visfatin, as shown by immunohistochemical analysis, is present in the cytoplasm of both SLCs and LLCs; a moderate signal was observed in the early, middle, and late CL, while a weak signal was observed in the regressed CL (corpus albicans) [336]. Visfatin expression at the gene and protein levels was also confirmed in the bovine CL [83]. Annie et al. noted the changing visfatin expression profile in the ovary during the estrous cycle in mice. Intense immunostaining in CL from proestrus and diestrus ovary and moderate from metestrus phases was demonstrated. Western blot analysis of the entire ovaries homogenates showed the highest expression of the adipokine in the proestrus phase and the lowest in diestrus. These reports indicate that visfatin can be involved both in the synthesis of P_4_ in the early stage of the luteal phase, as well as in the regression of CL from the previous cycle in proper time. Nevertheless, these assumptions require confirmation [337]. Visfatin mRNA and protein were also detected in the human luteinized Gc [85]. Unpublished research of our team on the porcine ovary also showed a variable expression profile of the visfatin expression. At the transcriptional level, we observed the highest abundance of *NAMPT* in the early CL, and the smallest in the middle CL. Conversely, at the protein level, the highest expression was observed in the mid-luteal CLs. Moreover, both LH and P_4_ in early and mid-luteal CL stimulated the expression of the visfatin protein and its release into the culture medium by LCs, while PGE_2_ and PGF_2α_ decreased them during the whole luteal phase. We also showed the expression of this adipokine at the early stages of pregnancy, especially during the maternal recognition of pregnancy and implantation, suggesting its important role in gestation maintenance at a very early stage. Additionally, our studies indicated an estrous phase-dependent effect of visfatin on P_4_ secretion. In the early and late luteal phases, visfatin reduced the secretion of this steroid. Interestingly, in cultures from the middle CL, it significantly increased P_4_ secretion. The mechanism of visfatin action remains to be studied.

Evidence for the involvement of visfatin in the proper functioning of luteal tissue is still growing. At the moment, it has been shown that, in the in vitro-cultured LCs of water buffalo, visfatin stimulated the secretion of P_4_. These changes are also evident in the increased amount of mRNA of steroidogenic enzymes involved in the synthesis of P_4_; *STAR*, *CYP11A1*, and *HSD3B1* [336]. In the human luteinized Gc, no effect of visfatin alone on the P_4_ and E_2_ secretion was observed. In contrast, the combined treatment of visfatin with IGF1 increased the secretion of these steroids compared to the action of IGF-1 alone; such an effect was not observed in the case of FSH stimulation. Additionally, visfatin stimulated IGF-1 induced the proliferation of the human Gc, and this effect was completely abolished by the pharmacological blocker of visfatin, FK866. Treatment with human recombinant visfatin rapidly also increased the phosphorylation of AKT, ERK1/2 kinases, and p38 protein [85].

### 4.5. Vaspin

Our previous research was the first to detect vaspin expression in the porcine CL; we showed that both, vaspin and GRP78 mRNA and protein level change during the estrous cycle, the highest expression was observed in the mid- and late compared to early luteal phases [338]. These changes were probably connected with fluctuating levels of sex hormones during the estrous cycle. This observation was followed by the in vitro studies which showed that LH, P_4_, PGE_2,_ and PGF_2α_ decreased vaspin protein expression and its secretion into the culture medium. Moreover, vaspin and GRP78 immunolocalization was shown in SLCs and LLCs cytoplasm [338], which was in agreement with our previous report, where we described vaspin expression in Gc and Tc [98].

All obtained data indicated vaspin’s role as a luteotropic factor in porcine CL. This adipokine, via the activation of GRP78 receptor and PKA, increased P_4_ secretion, as well as the expression of STAR protein and enzymes CYP11A1, HSD3B1 participating in cholesterol conversion to P_4_ [338]. Our research indicated a vaspin stimulatory effect on E_2_ secretion via the upregulation of CYP19A1 in porcine CL [338]. Interestingly, vaspin combined with LH decreased E_2_ secretion, and additionally stimulated the expression of CYP11A1 and HSD3B1, which is probably linked with the negative effect of LH on vaspin level in the LCs. Another study of our team showed also that vaspin, via GRP78 and MAP3/1, increased the PGE_2_/PGF_2α_ ratio, as well as the ratio of their receptors prostaglandin E receptor 1 (PTGER1)/PTGFR [338]. Another confirmation of luteotropic vaspin action was the stimulatory effect on angiogenic factors VEGFA, FGF2, ANG-1 secretion to the culture medium and mRNA expression in the LCs, as well as its stimulatory effect on proliferation and inhibitory on apoptosis via GRP78 and MAP3/1 [339]. Briefly, after 24h of the in vitro culture, vaspin downregulated caspase 3/7 activity, and caspase 3 expression, as well as Bcl-2-associated X protein (BAX)/B-cell lymphoma-2 protein (BCL-2) ratio, and elevated PCNA and cyclin A, markers of proliferation, levels.

### 4.6. Chemerin

Much less is known about the chemerin system expression in CL; the presence of transcripts and proteins of chemerin and its three receptors has been reported only in the CL of pregnant and non-pregnant pigs [340], and cattle [140]. Gene and protein expression of the adipokine and CMKLR1 has been observed in whole ovary lysates of rats [341,342]. The authors also reported the up-regulatory effects of 5α-dihydrotestosterone (DHT) [341] and obesity state [342] on the ovarian expression of chemerin and CMKLR1. Moreover, the presence of chemerin, CMKLR1, and GPR1 mRNAs, and GPR1 protein was noted in murine whole ovary lysates [133,343,344]. Some studies suggest that the expression of the chemerin system in CL may be dependent on the animal’s local hormonal milieu, including the levels of steroid hormones and prostaglandins [340,343].

Studies on the effect of chemerin on CL physiology are scarce and limited to only two animal species—mice and pigs. In mice, the research was conducted on two in vitro models [344]. In the murine superovulation model, where luteinization was induced by PMSG-hCG, P_4_ secretion in the luteal tissue culture was inhibited after chemerin administration. Moreover, P_4_ suppression was accompanied by the inhibition of gene expression of key steroidogenic enzymes (STAR, CYP11A1, and HSD3B1) [344]. In the same study, in the murine model of luteolysis induced by PGF_2α_, chemerin stimulated apoptosis and, consequently, luteolysis in the luteal tissue culture, by increasing the gene expression of caspase 3. It was also found that chemerin significantly inhibited P_4_ secretion by mice luteal tissue in this experimental model [344]. In turn, the administration of anti-GPR1 antibodies in both in vitro models completely abolished the observed effects of chemerin, which implies that GPR1 may be directly involved in the regulation of CL formation and luteolysis in mice [344]. In the in vitro study on pigs, chemerin has been shown to affect the basal and LH- and/or insulin-induced secretion of steroid hormones such as P_4_, androstenedione (A_4_), T, estrone (E_1_), and E_2_ by LCs during the early, mid-, and late luteal phases of the estrous cycle, as well as during early pregnancy, i.e., during the maternal recognition of pregnancy and beginning of implantation [345]. In this study, chemerin exhibited mainly a stimulatory effect on P_4_, an inhibitory on E_2_, and a differentiated effect (depending on the studied phase of the cycle/pregnancy) on A_4_, T, and E_1_ [345]. A subsequent in vitro study on pigs, conducted by our research team, also showed that chemerin modulated angiogenesis and apoptosis processes in LCs harvested during early, mid-, and late luteal phases of the estrous cycle [346]. The study demonstrated that chemerin stimulated the production of VEGFA and FGF and increased the protein abundance of angiogenic factors’ receptors, vascular endothelial growth factor receptors types 1, 2, and 3 (VEGFR1, VEGFR2, VEGFR3), and fibroblast growth factor receptors type 1 and 2 (FGFR1, FGFR2) in these cells [346]. The study also revealed that chemerin affected the protein abundance of apoptosis-related factors, i.e., first apoptosis signal (Fas) and Fas ligand (FasL), BCL-2, and caspase 3 in the porcine LCs [346]. Additionally, in the study on the global transcriptome of the porcine LCs collected from gilts during the mid-luteal phase of the cycle, the chemerin impact on genes whose protein products are engaged in the PGE_2_ (up-regulation of *cPLA2*, *PTGS2*, *COX2*, *PTGES,* and *PKIB*) and P_4_ synthesis pathways (down-regulation of *HSD3B1*) has been shown. Furthermore, it was found that chemerin influenced the expression of genes whose products are related to the regulation of the apoptosis process (up-regulation of caspase: *CASP10*, *CASP3*, *CASP7*, *BAK1*, *PMAIP1*, *CFLAR*, and *PFKFB3*), indicating the activation of both pro-survival and pro-apoptotic signaling pathways in the porcine LCs in the presence of chemerin [347]. Furthermore, the study demonstrated the effect of chemerin on the expression of many genes whose protein products are associated with the NFKB and JAK/STAT3 signal transduction pathways, which, in turn, are related to the processes of cell differentiation, proliferation, and migration, as well as the regulation of the apoptosis process [347].

### 4.7. Orexins

Until now, the luteal expression of all components of the orexin system, including *PPO*, *OX1R*, and *OX2R* transcripts, as well as OXA, OXB and both receptor proteins, have been described only in pig [162,165,348,349]. The presence of mRNAs and proteins of OX1R and OX2R has been reported in rat CL [159,350]. Transcripts of PPO and both OXRs have also been noted in the CL of water buffalo [351]. It was also observed that plasma concentrations of OXA in pigs were the highest in the early luteal phase (days 2 to 3 of the cycle), while the plasma concentrations of OXB were highest in the follicular phase (days 17 to 19) of the estrous cycle [158]. The concentration of *OX1R* mRNA in the porcine CL reached the highest values in the early luteal phase, decreasing in the following days of the estrous cycle, while the content of *OX2R* mRNA was the highest in Gc in the follicular phase [162]. Similarly, the concentration of OXA and OXB proteins in CL varied during the cycle, and was highest in the mid and late luteal phases, respectively [165]. The presented results suggest the clear effect of the hormonal status of animals on the expression of orexin system components.

The role of the orexin system in the development and degeneration of the luteal cells is poorly understood, and the results are not always conclusive. Basini et al. [349] hypothesized that OXA, acting locally in the ovary, may induce the regression of the CL because it inhibits in vitro P_4_ secretion by the porcine Lc and new vessel growth in the porcine aortic endothelial cell line. On the other hand, Grasselli et al. [352] showed a stimulatory effect of OXB on angiogenesis based on the same experimental model. In CHO cells transfected with OX1R or OX2R cDNAs, orexins caused cell growth inhibition and stimulated cell death by apoptosis [353,354]. In the rat LCs, both OXA and OXB decreased P_4_ secretion. Interestingly, the OXA effect was abolished only when both OX1R and OX2R antagonists were present in the culture medium, suggesting some complementary action between these two types of receptors. In the case of OXB influence on the steroid release, its effect was blocked only by the OX2R antagonist [350]. In other studies, using the porcine LCs, it was found that there was either an inhibitory effect of OXA on P_4_ production [349] or that there was a lack of effect of both orexins [165]. The observed inhibitory effect of OXA was probably due to a reduction in the expression of the *CYP11A1* gene [349]. The inhibition of P_4_ production, coupled with the negative effect of OXA on ovarian angiogenesis and induction of programmed cell death mentioned earlier, may suggest a potential involvement of OXA in luteolysis. The influence of orexins on steroidogenesis was also noticeable in follicular Gc, the precursor cells of large luteal ones. Contrary to LCs, P_4_ secretion by Gc was stimulated by OXA in rats [355] and sheep [356,357]. The increase in P_4_ secretion was associated with the increased expression of STAR protein, HSD3B1, and CYP11A1, under the influence of OXA [355,356,357], as well as by down-regulating bone morphogenetic protein (BMP) signaling [355]. Moreover, FSH-induced E_2_ secretion by the porcine Gc harvested from the preovulatory follicle was suppressed by OXA and OXB [165]. It, therefore, seems that the role of orexins in CL differs depending on its stage of development: during the transformation of follicular cells into LLCs, orexins stimulate luteinization, while fully developed CL responds to OXA by inhibiting P_4_ synthesis, which is an element of luteolysis.

### 4.8. Ghrelin

Studies show a variable expression of ghrelin and its receptor GHSR in different species depending on the stage of development of the CL. Increased levels of ghrelin were observed in the LCs from the mid- and late luteal phase in buffaloes [358], rats [359], pigs [206,360], goats [361], horses [297], sheep [362], and humans [240,363]. In mature pigs, a significant increase in the level of ghrelin during the estrous cycle was demonstrated. Interestingly, the greatest rise in ghrelin protein expression was noted in the late luteal phase, which was confirmed by immunohistochemical analysis, showing the local presence of ghrelin in the cytoplasm of LLCs. However, a lack of the functional ghrelin receptor GHSR1a expression was observed during the development of the CL in pigs [360]. Additionally, the increased mRNA and protein expression of ghrelin was demonstrated in rats during the luteal phase. In contrast, lower levels of ghrelin during the CL formation and regression phase have been found. Similarly, it was confirmed by immunohistochemical analysis, showing the expression of ghrelin in the cytoplasm of steroidogenic LCs. Despite the significantly increased expression of ghrelin in CL, the plasma concentrations of this peptide during the estrous cycle of rats were not differentiated [359]. Among others, Tropea et al. observed the mRNA expression of the ghrelin receptor in the human LCs [363]. What is important is that immunohistochemical analyses showed a lack of ghrelin expression in the human ovarian follicles or CL in the early stages of CL development. Ghrelin immunoreactivity in the mid- and late luteal phases has been demonstrated. On the other hand, GHSR1a expression has been shown in a wider ovarian cell spectrum, i.e., in oocytes and at all stages of CL development, and in interstitial hilus cells [240]. Interesting research by Galvão et al. confirmed the increased level of GHR/GHSR1A in the later CL phase in the equine ovary [297]. The expression of ghrelin and its receptor in the whole reproductive system in Holstein heifers, including the CL, has been demonstrated. It has been observed that ghrelin is present in the cytoplasm and GHSR in the luteal cell membranes in cattle [364]. Caminos et al. [359] observed similar levels of ghrelin expression in the ovaries of pseudopregnant rats compared to cyclic animals. In contrast, ovarian monitoring showed a decrease in ghrelin expression in the later stages of rat gestation. Physiologically, CL is the most functional in the first weeks of pregnancy. In later stages, it gradually regresses, and the placenta overtakes the secretory function. The study results suggest that ghrelin expression is directly proportional to CL activity in cyclic and gestational rats [359]. Other studies showed higher levels of ghrelin expression during early pregnancy than the estrus cycle in the buffalo [365].

A convergent profile of ghrelin expression and P_4_ secretion was demonstrated, confirming the important role in the regulation of CL development and functioning in rats [359]. One of the first studies determining the functionality of ghrelin in the human LCs confirmed its inhibitory effect on ovarian function. The inhibition by ghrelin of both basal and hCG-induced P_4_ secretions in human LCs was demonstrated [363]. Similarly, in the study of the Rak-Mardyła group, ghrelin had an inhibitory effect on P_4_ secretion and HSD3B protein expression in cultured porcine LCs [360]. Ghrelin reduced the release of PGE_2_ but increased the release of luteolytic PGF_2α_ [363]. Moreover, ghrelin led to an increase in PGF_2α_, nitrite, and TNFα in the mid-stage of CL development, promoting luteolysis in the mare [297]. Romani et al. observed that the non-acylated form of ghrelin may affect the regulation of luteal steroidogenesis and the reduction of P_4_ and VEGF release in humans [366]. Treatment of LCs culture with ghrelin led to a decrease in P_4_ concentration and mRNA expression of *HSD3B1* and *CYP11A1* [358]. Interestingly, the administration of a GnRH antagonist to inhibit ovulation and CL formation led to a reduction in the mRNA expression of ghrelin [359]. Studies have shown a correlation between the decrease in LH concentration following the administration of a GnRH antagonist and the expression of ghrelin in male rats [367]. However, the direct dependence on ovarian function requires further research. Research by Kheradmand et al. [368] showed that the treatment with ghrelin for 9 and 14 days had an anti-apoptotic effect in rat LCs throughout reducing the level of BAX protein and increasing the level of BCL-2 protein in rats. Ghrelin did not affect the level of caspase-3. Additionally, it was observed to increase the expression of the PCNA peptide associated with proliferation [368]. Ghrelin expression has been demonstrated in the cytoplasm of luteal regression cells in women with PCOS. Moreover, immunostaining revealed the presence of proliferation marker Ki 67 at the sites where ghrelin was expressed [369]. Research by Alirezaei et al. [370] focused on apoptosis and proliferation in pregnant sheep; immunohistochemical analysis showed high PCNA and BCL-2 expression at the fourth month of gestation in CL. However, in the 5th month of pregnancy, the level of caspase-3 increased. An increase in ghrelin concentration was correlated with the concentration of E_2_ in the ovarian follicles. In contrast, the authors reported an increase in P_4_ in CL around the 4th month of pregnancy. These studies indicate a regulatory role of ghrelin, mainly by E_2_ during pregnancy [370].

### 4.9. Kisspeptin

In 2006, Castellano et al. [371] found Kiss1 in rat CL. They found that Kiss1 was expressed in rat cyclic ovary and weakly expressed in corpus hemorrhagicum. The intensity of Kiss1 expression correlates with the stage of CL formation. Kisspeptin is strongly expressed in the steroidogenic cells of CL during proestrus, and there is a decrease in its expression when the CL is in regression [371]. Peng et al. found that 7 days after hCG treatment of PMSG-primed rats, there was an increase of Kiss1 and its receptor Kiss1R in CL. Indeed, Kiss1 and Kiss1R are expressed dominantly in CL compared to Gc and Tc [372,373]. Cielesh et al. [374] found Kiss1 and Kiss1R in the bitch ovary. Kiss1 is expressed in CL, only in the lateral marging of the cyclic bitch. In contrast, Kiss1 is not expressed in the CL of the prepubertal, anestrous bitch. The Kiss1R is expressed in CL during all of the stages of the reproductive cycle [374]. Tanyapanyachon et al. [375] found that Kiss1 is expressed in cat CL, mainly at the formation and development stages, but there is no expression in steroidogenic cells. At the formation stage, Kiss1 is expressed both in the periphery and in the center of CL. In contrast, during the development stage, Kiss1 is more expressed in the periphery than in the center of CL. Kiss1R is expressed in the cytoplasm of LCs in the periphery and the center of CL during formation and development [375]. Mishra and al. found Kiss1 and Kiss1R in buffalo LCs from developing CL [376] and in the cytoplasm of LLCs from early and middle CL [377]. Maranesi and al. [378] found the Kiss1 system in rabbit CL. Kisspeptin is expressed in nuclei and cytoplasm of LCs from CL during early (Day 4), mid- (Day 9), and late (Day 13) pseudopregnancy. The Kiss1R is expressed in the cytoplasm of LCs mostly during early pseudopregnancy but it is also expressed during mid-pseudogestation but not during the late stage. The Kiss1 system could be involved in CL protection from luteolysis. It was the first study about the Kiss1 system involvement in the CL lifespan of rabbits by modulating P_4_, prostaglandins synthesis, and prostaglandin synthase 2 (PGS2) expression during pseudopregnancy [378].

Inoue et al. [379] observed that, when musk shrews are treated with suncus kisspeptin consisting of 29 amino acid residues (sKp-29), CL is formed 3 days after the treatment, as well as after coupling. This effect is blocked by a pre-treatment with a GnRH antagonist. This suggests that Kiss1 stimulates GnRH release in the hypothalamus. It was already reported in spontaneous ovulating species. So, sKP-29 acts as a coupling stimulus to induce suspected ovulation [379]. In rats, it was shown that Kiss1 antagonist P234 administration into ovarian bursa for 3 days from the morning of the proestrus day can induce changes in CL morphology. Indeed, the outer layer of the CL was not well-formed [373]. In rat LCs, Kiss1 increases P_4_ secretion. Moreover, Kiss1 and hCG together increase P_4_ secretion more than hCG or Kiss1 alone. So, Kiss1 could favor CL formation and the sustainable development of pregnancy by stimulating key steroidogenic enzymes and proteins like STAR and CYP11A1. Finally, Kiss1 could be a luteinization stimulator and a regulator of CL lifespan by acting on steroidogenic enzymes which modulate P_4_ production [372]. In mice, Stephens et al. generated the transgenic mice lacking PGR exclusively in kisspeptin cells (KissPRKO). It is mice knock-out for the P_4_ receptor in kisspeptin-positive cells. They found that KissPRKO female mice have less CL as a result of a decrease in ovulation rate. The E_2_ administration did not beget LH surge, so they hypothesized that the infertility of KissPRKO female mice is due to a decrease of neuronal activation with an absence of GnRH pulse induced by Kiss1 [380]. In luteal explants from buffalo, Kiss1 increased the expression of genes regulating the synthesis of P_4_ in CL as *STAR*, *CYP11A1,* and *HSD3B1* and luteinizing hormone receptor (*LHR)* 12h after treatment, whereas it decreased 24h after treatment. In contrast, Kiss1 enhanced a hCG-induced decrease in genes expression. So Kiss1 could have a potential role in steroidogenesis in buffalo [377]. In the rabbit, the agonist Kiss-10 increased P_4_ secretion by CL in early and mid-pseudopregnancy. In contrast, the antagonist Kiss-234 had the opposite effect [378]. In the rabbit, the agonist Kiss-10 decreased PGF_2α_ secretion in early and mid-pseudopregnancy. In contrast, the antagonist, Kiss-234, has the opposite effect. In addition, Kiss-10 increased PGE_2_ secretion and Kiss-234 had the opposite effect. It improved P_4_ synthesis during the early CL stage and did not affect PGF_2α_ and PGE_2_ secretion at the mid- and late luteal stages. Kiss-10 and Kiss-234 did not affect prostaglandin-endoperoxide synthase 1 (PTGS1). In contrast, Kiss-10 decreased the luteal activity of PTGS2 and Kiss-234 increased it during early and mid-pseudopregnancy. Thus, it seems that Kiss-10 positively regulates P_4_ synthesis. Kiss1 has a luteotropic role and increases luteal P_4_ production, maybe via autocrine and/or paracrine mechanisms inducing a decrease of PGF_2α_ and then an increase of PGE_2_ due to the blocking of PTGS2 activity [378]. No data are available about the effect of Kiss1 on angiogenesis in CL. However, in bat ovary treated with a low dose of Kiss1 (100 ng/mL), there was an increase in VEGF protein, which is a biomarker of angiogenesis. With a high dose of Kiss1 (1 µg/mL), the opposite effect is observed [381]. Furthermore, in the same study, it has been shown that Kiss1 increased the expression of PCNA simultaneously decreasing caspase 3; when P_4_ is added to Kiss1, the ovarian cells proliferation is higher [381].

### 4.10. Phoenixin

So far, the expression of phoenixin in the CL has only been documented in the human ovary; immunohistochemical analysis showed an increase in phoenixin-20 and GPR173 expression with follicle growth, so that the most intense signal was obtained in the antral follicles and CL [276]. Our preliminary unpublished studies also indicate the presence of phoenixin-14/GPR173 in the porcine CL. Both the mRNA of *SMIM20* and the level of the phoenixin-14 protein increase during the luteal phase. On the other hand, the expression of the *GPR173* gene reaches the highest level in the early luteal phase and then drops, while the protein level increases during the luteal phase. We also localized phoenixin-14 in the cytoplasm and GPR173 in the cell membrane, mostly in LLCs. Additionally, the secretion of phoenixin by LCs can be downregulated by LH, P_4_, and prostaglandins E_2_ and F_2α_ in the mid-luteal phase.

The influence of phoenixin on ovarian physiology is still poorly understood. So far, in the literature, we could not find any information about the influence of phoenixin on the functions of LCs. It remains still undiscovered. Nevertheless, with the growing research on this peptide, it is only a matter of time to discover its other beneficial aspects on reproduction, especially considering the growing evidence of phoenixin involvement in steroidogenesis, Gc proliferation, and oocyte maturation [382]. Indeed, in vitro experiments on the human non-luteinized Gc line (HGrC1) showed that phoenixin-20 significantly increases proliferation, the secretion of E_2_, and the expression of CYP19A1, LHR, and FSH receptor (FSHR), as well as increasing the number of ovulated mouse oocytes with a higher level of maturation [276]. Similarly, in zebrafish, the same isoform of phoenixin stimulates steroidogenesis via an increase of the expression of steroidogenic enzymes like *cyp11a1*, cytochrome P450 family 17 subfamily A member 1 (*cyp17a1*), *cyp19a1, hsd17b*, and estrogen receptors *esr2a* and *esr2b* in the ovary. In addition, phoenixin-20 has been observed to be involved in vitellogenesis by increasing the transcript level of hepatic vitellogenin and promoting egg maturation [288]. Our preliminary study indicated that phoenxin-14 can dose-dependently regulate the secretion of P_4_ and E_2_ in porcine CL. Nevertheless, further research is needed to confirm this effect, as well as to understand other aspects of the role of phoenixin in the formation, functioning, and regression of the CL. It is worth adding that phoenixin-14 contains aa sequences present in the structure of MITRAC7, a protein responsible for the proper functioning of the mitochondrial respiratory chain. In addition, the MITRAC complex consists of various COX1 complexes, which in turn participate in the synthesis of prostaglandins. The relationship between phoenixin and prostaglandin synthesis has not been understood so far, but in the light of this information, it seems that it is worth developing this question in the future [383,384].

## 5. Involvement of Adipokines and Neuropeptides in Corpus Luteum Pathology

**Figure 3:**
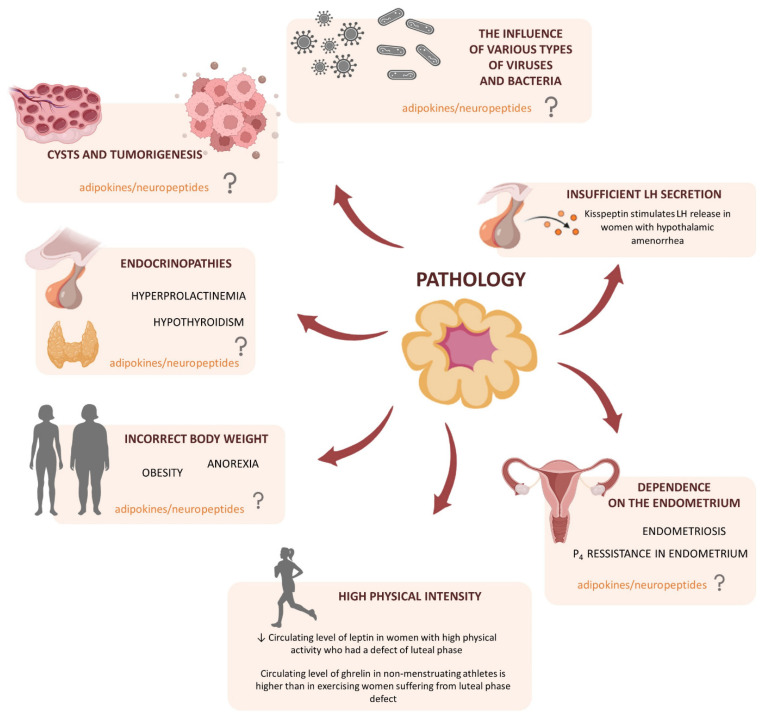
Involvement of adipokines and neuropeptides in corpus luteum pathology. P_4_—progesterone, LH—luteinizing hormone, ↓—decrease, ?—unknown effect.

The corpus luteum can be affected by numerous pathological conditions, including, for example, relatively frequently observed cysts [385]. Their rupture is a serious problem both during the menstrual cycle, by temporarily stopping menstruation, and pregnancy, where it can contribute to the loss of a pregnancy due to abnormal embryo invasion and ectopic pregnancy [386]. Moreover, this ailment is very often noted in the period after the first menstruation, contributing to the disturbance of the ovulation process [387]. The main symptoms include persistent abdominal pain in 84.6% and acute abdominal pain in 15.4% of patients [387]. Interestingly, their detection, and thus, the diagnosis itself, is difficult, due to the relatively small size and fairly thick walls. In addition, other studies investigated that the greatest cyst production mainly occurs immediately after CL formation [388]. In turn, the early formation of cysts is attributed, among others, to premature closure of the ovulation site [389]. Furthermore, recent studies suggest that the CL cysts can very often, in as many as 80% of cases, be confused with ectopic pregnancy [390]. Data indicate that, in patients with positive serum β-hCG levels, a significant resistive index of greater than 0.7 for an adnexal mass may be important in differentiating ectopic pregnancy from CL cysts [390]. In addition, studies on cattle have shown that age and milk-producing capacity may be criteria for a higher risk of luteal cysts—older cows and those with a higher production rate are more likely to develop cysts [391]. The detection of cysts may be based on rectal palpation and ultrasonography combined with the measurement of serum P_4_ concentration [392]. In the case of the problems posed by follicular cysts, an increased secretion of Kiss1 has been observed in cows. These results were connected with direct or also indirect with an increase in LH secretion [393]. In addition, other studies have demonstrated the immune reactivity of Kiss1 and the Kiss1R in LCs at both the nuclear and cytoplasmic levels [378]. It was investigated that the Kiss1 system is expressed in the CL of pseudopregnant rabbits and presents a luteotropic effect via the downregulation of PTGS2, which in turn, reduces PGF_2α_ levels and increases PGE_2_ and P_4_ [378] (Figure 3).

Additionally, CL disorders may result from the influence of various types of viruses, and bacteria on the proper functioning of the immune system, which manifests in the form of inflammation [385]. Interestingly, processes related to the immune system are often associated with angiogenesis [394,395]. Research shows that in rats, cattle, and humans, following the process of ovulation in the area of luteinizing, developing CL, there is a high number of immune cells, macrophages, and eosinophils. These kinds of cells are responsible for the secretion of angiogenic factors: VEGF, FGF, and hypoxia-inducible factor 1 (HIF-1) which may affect inflammatory-induced angiogenesis in the early luteal phase. Such data suggest that the immune system may play an important role in early luteal development [396,397,398]. It has also been reported that luteal cells inflammation can be induced by the herpes virus [399]. The time of exposure to the virus during the estrous cycle has been investigated to be important in the formation of inflammation which infiltrates the ovary, with CL being one of the most unfavorable changes [400]. As a consequence of viral exposure, i.a., a reduction in P_4_ production by CL is noted, with plasma levels only around 2 ng/mL [385,401]. Bacteria can also be another cause of ovarian dysfunction. Studies conducted on cows show, i.a., that the intrauterine infusions of *Trueperella pyogenes* caused luteal dysfunction [402]. Moreover, uterine bacterial infection after delivery may also contribute to premature loss of CL [403]. It has been shown, i.a., that a consequence of postpartum uterine infection with bacteria is that later developing CL secretes much less P_4_ than in non-infected individuals [404]. It has been investigated that *Escherichia coli* bacteria can affect the development of diseases of the uterus of dairy cows due to specific virulence factors, e.g., fimH, hlyA, or astA, and the activation of this inflammation is the basis for the development of endometrial and embryo damage, and delayed ovulation, as well as shortening or extension of the luteal phase [405]. At the moment, there are no data on the influence of individual adipokines and neuropeptides in the inflammatory processes of the ovary and of CL itself, which introduces a wide perspective for future research in this area. It is worth noting here that previous studies indicate the participation of adipose tissue hormones in inflammatory processes in women with one of the most common pathologies of the ovary, namely PCOS. For example, literature data indicate that, if the hypothalamus has a low-grade chronic inflammatory state, it may be a cause of central leptin resistance in PCOS rats [406]. Additionally, it was also investigated that the adiponectin/leptin ratio could serve as a potential biomarker of low-grade inflammation in women with this syndrome. Unfortunately, the scope of research on other adipokines in this type of inflammatory process is still very limited [407].

Moreover, in the case of abnormal function of this organ, neoplasia is also noted. For example, studies have shown that lymphosarcoma can occur in the ovary and, above all, with particular risk in CL [408,409]. In addition, it was investigated that, due to relatively high blood flow and well-developed capillaries, tumors of other organs also have the possibility of metastasis to CL [389]. The role of adipokines in tumorigenesis in CL has not yet been investigated. However, the latest reports show that apelin can stimulate the survival of cancer ovarian cells, which makes the apelinergic system a potential target during therapies eliminating spreading metastases [410]. In addition, leptin appears to be another adipokine contributing to a worse prognosis in ovarian cancer. Interestingly, research conducted on a group of obese women has shown that adipokine promotes cancer progression by stimulating the migration and invasion of neoplastic cells [411]. It was shown that adipokine can reduce the proliferation of the type of cells caused by bisphenol and its derivatives [412]. The present literature data indicate the possibility of adipokines’ involvement in the ovarian structure such as CL, but more extensive research is needed on this matter.

During the correctly occurring luteal phase, P_4_ is produced, which determines the proper proliferation of uterine cells in order to prepare this organ for a possible pregnancy. Thus, the basic problem posed by the dysfunction of the luteal phase is the dysregulation of the menstrual cycle, and thus the increased risk of problems with becoming pregnant or maintaining the pregnancy itself. Interestingly, it has been shown that, in patients with luteal phase deficiency, the menstrual cycle lasts 24.2 days, while in healthy women, it is about 29 ± 3 days [413]. Moreover, defects of the luteal phase occur when the phase lasts less than 10 days [414]. There is a number of potential causes contributing to this disorder, one of which is anorexia nervosa (AN). Research from 2016 indicates that around 75 million girls are seriously underweight worldwide [415]. Despite the fact that dysfunction of the HPO axis may be reversible in women with AN, unfortunately, the consequences of this disease affect female fertility and cause pregnancy complications. The cause of them is the low level of P_4_ observed in patients with AN, indicating a disturbance in the luteal phase responsible for the production of the hormone [416]. Interestingly, it has been shown that the luteal phase defect is observed with a 10–15% weight loss [413]. Obesity also negatively affects the course of the luteal phase and related disorders of reproductive function [417]. In both anorexic and obese women, there are changes in the amplitude of LH pulses and a decrease in the excretion of the main P_4_ metabolite, namely pregnanediol glucuronide, in the luteal phase, which could indicate the defect [417,418]. It has been investigated that both weight gain and weight loss induce changes in the CL transcriptome [419]. Moreover, in the same study, it was shown that obesity impairs P_4_ secretion in the middle luteal phase, which is related to the downregulation of CL steroid pathways [419]. However, correlation studies between the levels of circulating KISS1, adiponectin, and leptin during the early follicular, pre-ovulatory, and luteal phases in women of normal body weight and overweight did not indicate substantial differences in adipokine levels between the groups [420]. In turn, another cause of a defect in the luteal phase may be increased by exercise, and the absence of regular periods may affect 1 to 46% of women [421]. Previous studies have shown that, in the case of vigorous physical activity, both anovulatory cycles and a defect in the luteal phase are the most common disorders of the menstrual cycle [422]. A luteal phase defect manifested by a reduction in P_4_ production, and thus insufficient endometrial stimulation, is also associated with a disturbance in oocyte maturation, which, in this case, reduces reproductive capacity, and leads to premature loss of pregnancy [423]. It seems that changes in the adipose tissue mass and the adipokines that it produces may contribute to the observed disorders in women of high physical intensity. Research conducted on a group of young female basketball and handball players preparing for the competition showed a statistically significant reduction in the level of circulating ghrelin and leptin during moderate and long-term aerobic exercise and an increase in visfatin concentration after short-term exercise. In addition, a significant increase in the level of adiponectin was also observed as a result of speed and conditioning training [424]. Interestingly, a study conducted on women with high physical activity who had a defect in the luteal phase showed a significant reduction in the level of circulating leptin, however, this result could be directly related to the reduction of body adipose tissue in this group of patients [425]. Moreover, other studies have also indicated that ghrelin levels in non-menstruating athletes are higher than in exercising women suffering from a luteal phase defect, which also suggests a relationship between adipokine levels and reproductive capacity [426]. Furthermore, it is worth noting that the problem of hypothyroidism may also be indirectly associated with anovulatory cycles and a defect in the luteal phase [427]. Moreover, hypothyroidemia is also observed in highly active women [428]. In addition, some studies indicated that the treatment of infertility (caused by a luteal phase defect) by thyroid hormones may increase the fertilization rate in patients before undergoing hormone therapy with chorionic gonadotropin [429]. The problem of hypothyroidism may also be indirectly associated with anovulatory cycles and a defect in the luteal phase [427]. Additionally, it was investigated that, in actively training women, a decrease in the level of circulating leptin positively correlates with a downstream of thyroid hormones [430]. There is some evidence that a defect in the luteal phase may also be related to the course of endometriosis. It has been investigated whether, in infertile women with endometriosis, the defect of the luteal phase is manifested in the form of dysfunction of small and large LCs, and thus LH-dependent P_4_ production [431]. However, some research is in opposition to linking endometriosis with a defect in the luteal phase. The study diagnosing a luteal phase defect, conducted on 84 patients, did not show a statistically significant correlation between endometriosis and the same type of CL dysfunction [432]. In addition, previous histological examinations of the endometrium also showed no significant differences in the levels of P_4_ receptors in women with a luteal phase defect compared to healthy patients [433].

Interestingly, the defect in the luteal phase may be caused by the disturbance of the secretion of PRL by the endometrium. It has been shown that, in women with a luteal phase defect, the endometrium produces less PRL, suggesting that tissue levels of this hormone may aid in the diagnosis of a luteal phase defect [434]. In addition, studies conducted on a group of healthy women and with endometriosis have shown that sick women are characterized by significant hyperprolactinemia in the mid-luteal phase. These results may support the belief that hyperprolactinemia may interfere with luteal function, causing infertility in patients with endometriosis [435]. It has also been shown that leptin has the ability to increase the production of PRL via endometrial stromal cells [436], however, there is no evidence that this could directly affect the luteal phase defect.

It is worth noting here that the spectrum of known luteal pathologies of CL is extensive, and may be connected with abnormal P_4_ secretion as well as persistent CL or premature luteolysis. In the first case, impaired P_4_ production may be caused by the problem of persistent CL, as well as by inappropriate follicles development prior to ovulation or abnormal luteal maturation [437]. Dysfunction, such as persistent CL, may result from endometrial hypoplasia or the intra-uterine injection of irritating solutions [408,438]. In turn, premature luteolysis can be directly combined with the injection of specific hormones on different days of the cycle. It has been shown that exposure to oxytocin or P_4_ in the early days of the estrous cycle and to E_2_ during pregnancy may cause premature luteolysis [408,439,440,441]. In addition to disturbed P_4_ secretion, the clinical picture of luteal phase deficiency may also be caused by an inadequate response of the endometrium to the correct concentration of steroid hormones, and the main cause is endometriosis, as well as PCOS [442]. In endometriosis, persistent inflammation may be the cause of this condition [443], while one of the main reasons of P_4_ resistance in women with PCOS is the decreased expression of P_4_ receptors in the endometrium [444].

The cause or the common denominator of many of the above-mentioned pathologies of the CL and the associated defects of the luteal phase is the insufficient secretion of P_4_. Both adipokines and neuropeptides may prove helpful in the treatment of luteal phase dysfunction caused by a deficiency in P_4_ secretion. The methods currently used in assisted reproduction treatment consist of, apart from the exogenous administration of P_4_, the use of various stimulants of the endogenous secretion of this steroid. One of the most common is the administration of hCG, which is to induce an increase in P_4_ secretion, but also the GnRH agonists indirectly influencing P_4_ biosynthesis, by stimulating the LH surge [442]. Here, a key role may be played by neuropeptides such as kisspeptin, of which the research has shown to be a potent LH stimulant. A strong stimulation of LH release, especially in the preovulatory phase, has been observed in healthy women who were subcutaneously injected by various doses of kisspeptin 54 [445]. Similarly, Jayasena et al. observed that subcutaneous administration of kisspeptin 54 strongly stimulates LH release in women with hypothalamic amenorrhea [446]; a similar effect on LH secretion was observed in phoenixin [277]. Nevertheless, further research and analyses are necessary to determine the direct involvement of adipokines in the CL pathologies we discussed earlier, i.e., the formation of cysts, inflammations, adhesions, neoplasia, or luteal dysfunction.

## 6. Conclusions

To summarize, literature data clearly indicated the involvement of different adipokines and neuropeptides on CL physiology, including its formation and regression, as well as the regulation of its main function, P_4_ synthesis. Adipokines: leptin, adiponectin, apelin, visfatin, vaspin, chemerin, and neuropeptides: orexins, ghrelin, kisspeptin and phoenixin are expressed in LCs, dependent on the luteal phase stage in different species, including human, rodent, and agricultural species. Moreover, its expression is modulated by the main hormones regulating luteal cells function, such as P_4_, LH, or prostaglandins. Its effect on CLs physiology depends mostly on the studied adipokine or neuropeptide, e.g., leptin, apelin and vaspin stimulates P_4_ synthesis, while adiponectin has an opposite effect. Actions of presented adipokines/neuropeptides may also be species-dependent, e.g., chemerin in mice decreases P_4_ level and stimulates the apoptosis of LCs, while in porcine, it stimulates steroidogenesis and angiogenesis. The observed effect may be also dependent on the development stage of CLs, as noted for orexins; during the transformation of follicular cells into LLCs, orexins stimulate luteinization, while a fully developed CL responding to OXA is connected to luteolysis. Nevertheless, adipokines and neuropeptides are important regulators of CLs physiology, and proper balance between them is needed to maintain CLs function. As shown for kisspeptin, mice with its knockout have fewer CLs as a result of a decrease in ovulation rate. What is more, a lot of work describing the complicated molecular mechanisms of adipokines action in CLs gives the knowledge necessary to modulate CLs physiology useful in the synchronization of the estrous cycle in female animals, and may improve their fertility. Moreover, adipokine and/or neuropeptide levels in the blood serum in the future may also be predictors of different pathological stages in CLs, as was described previously for different adipokines and ovarian follicles pathology PCOS [447]. For example, low levels of adipokines, which stimulate P_4_ secretion and maintain CLs function like leptin or vaspin, may indicate luteal phase deficiency. Future studies are necessary to determine the direct involvement of adipokines in CLs pathology, such as the formation of cysts, inflammations, adhesions, neoplasia, or luteal dysfunction, and the connection between these pathologies and adipokines blood level, which introduces a wide perspective for future research in this area, and makes adipokines specific markers of LCs dysfunction.

## Figures and Tables

**Table 1:** Expression of adipokines, neuropeptides and their receptors in corpus luteum during the estrous cycle. The factors that are expressed not only in the estrous cycle (EC) but also in the pregnancy (PR) corpus luteum have been appropriately marked. AdipoR1—adiponectin receptor 1, AdipoR2—adiponectin receptor 2, APJ—apelin receptor, CMKLR1—chemokine-like receptor 1, GPR1—G protein-coupled receptor 1, CCRL2—C-C motif chemokine receptor-like 2, OX1R—orexin receptor type, OX2R—orexin receptor type 2, GHSR—GH secretagogue receptor, KissR1—kisspeptin receptor, GPR173—G protein-coupled receptor 173.

Adipokine	Species Expression	
	Gene	Protein
leptin	human, rat, pig (EC, PR), cattle, horse, goat, water buffalo, dog (EC, PR)	human, rat, pig (EC, PR), cattle, horse, goat, water buffalo
LEPR	human, baboon (PR), cattle (EC, PR), dog, rat, pig (EC, PR), horse, water buffalo	rat, pig (EC, PR), horse, water buffalo, mouse, rabbit, alpaca, Japanese black bear
adiponectin	rat, cattle, pig, water buffalo	rat, cattle, pig, water buffalo
AdipoR1, AdipoR2	rat, cattle, pig, water buffalo	rat, cattle, pig, water buffalo
apelin	cattle, rhesus monkey, pig, mouse	cattle, sheep, pig, dog
APJ	cattle, rhesus monkey, pig, mouse	cattle, sheep, pig, dog
visfatin	water buffalo, cattle, pig (EC, PR)	water buffalo, cattle, mouse, pig (EC, PR)
vaspin	pig	pig
chemerin	pig (EC, PR), cattle, rat, mouse	pig (EC, PR), cattle, rat
CMKLR1	pig (EC, PR), cattle, mouse, rat	pig (EC, PR), cattle, rat
CCRL2	pig (EC, PR), cattle	pig (EC, PR), cattle
GPR1	mouse, pig (EC, PR), cattle	mouse, pig (EC, PR), cattle
orexin	pig, rat, water buffalo	pig, rat
OXR1, OXR2	pig, rat, water buffalo	pig, rat
ghrelin	pig, rat (EC, PR), horse, cattle, water buffalo, goat, sheep	pig, rat (EC, PR), human, horse, cattle, water buffalo, sheep
GHSR	pig, human, horse, cattle, water buffalo, goat	pig, human, horse, cattle, water buffalo,
kisspeptin	rat, water buffalo	rat, water buffalo, dog, cat, rabbit (PR)
Kiss1R	rat, water buffalo	rat, dog, cat, water buffalo, rabbit (PR)
phoenixin	pig	pig, human
GPR173	pig	pig

## Data Availability

Not applicable.
